# Reducing the influence of intramodular connectivity in participation coefficient

**DOI:** 10.1162/netn_a_00127

**Published:** 2020-04-01

**Authors:** Mangor Pedersen, Amir Omidvarnia, James M. Shine, Graeme D. Jackson, Andrew Zalesky

**Affiliations:** The Florey Institute of Neuroscience and Mental Health, The University of Melbourne, Melbourne, Victoria, Australia; The Florey Institute of Neuroscience and Mental Health, The University of Melbourne, Melbourne, Victoria, Australia; Brain and Mind Center, The University of Sydney, Sydney, New South Wales, Australia; The Florey Institute of Neuroscience and Mental Health, The University of Melbourne, Melbourne, Victoria, Australia; Department of Neurology, Austin Health, Melbourne, Victoria, Australia; Department of Psychiatry, Melbourne Neuropsychiatry Centre, The University of Melbourne, Victoria, Australia

**Keywords:** Complex networks, Graph theory, Participation coefficient, fMRI, Airports, *C. elegans*

## Abstract

Both natural and engineered networks are often modular. Whether a network node interacts with only nodes from its own module or nodes from multiple modules provides insight into its functional role. The participation coefficient (*PC*) is typically used to measure this attribute, although its value also depends on the size and connectedness of the module it belongs to and may lead to nonintuitive identification of highly connected nodes. Here, we develop a normalized *PC* that reduces the influence of intramodular connectivity compared with the conventional *PC*. Using brain, *C. elegans*, airport, and simulated networks, we show that our measure of participation is not influenced by the size or connectedness of modules, while preserving conceptual and mathematical properties, of the classic formulation of *PC*. Unlike the conventional *PC*, we identify London and New York as high participators in the air traffic network and demonstrate stronger associations with working memory in human brain networks, yielding new insights into nodal participation across network modules.

## Author Summary

It is challenging to reliably quantify how single elements (i.e., nodes) in a network are connected to different subcomponents (i.e., modules) of a network; this is important as intermodular connectivity contribute to efficient and distributed information processing. Participation coefficient (*PC*) calculates how distributed nodes are across modules. But *PC* is influenced by modularity algorithms that tend to favor large modules with strong intramodule connectivity, that in turn generate low *PC* values, even if a node has strong intermodule connectivity. We use a network randomization approach and show that by reducing the influence of intramodular connectivity, we obtain node participation results unaffected by size and connectedness of modules. This provides network scientists with new insights into the intermodular connectivity configurations of complex networks.

## INTRODUCTION

Many natural and engineered networks are modular. Networks that are highly modular can be partitioned into communities of nodes, or modules, such that the density of connections is greater between the nodes within modules, relative to the density between nodes in different modules. Some nodes have connections that are distributed across many modules, whereas others are only connected with other nodes in their own module. This distinction can provide important insight into a node’s functional role in a modular architecture.

A node’s intermodular connectivity is typically quantified with the participation coefficient (*PC*) (Guimerà & Amaral, 2005). *PC* provides insights into how specific nodes communicate between modules in a range of real-world networks, including air traffic and brain networks (Bertolero, Yeo, Bassett, & D’Esposito, 2018; Guimerà, Mossa, Turtschi, & Amaral, 2005; Kim & Kaiser, 2014; Pedersen, Zalesky, Omidvarnia, & Jackson, 2018; Power, Schlaggar, Lessov-Schlaggar, & Petersen, 2013; Sethi, Pedersen, & Jackson, 2016; Shine et al., 2016; Sohn, Choi, Ahn, Lee, & Jeong, 2011; Towlson, Vértes, Ahnert, Schafer, & Bullmore, 2013). To compute a node’s *PC*, the proportion of a node’s connections to each module is first determined, yielding a proportion for each module. These proportions are then squared, summed across all modules, and the resulting summand is subtracted from one to yield the node’s *PC*. *PC* of zero indicates a node that only connects with other nodes in its own module, whereas nodes with connections that are uniformly distributed across all modules have a *PC* of one.

**Figure 1.**
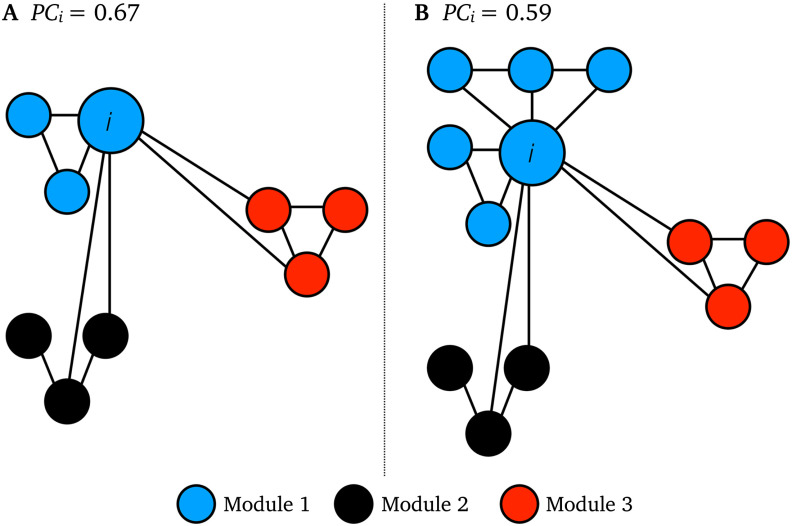
Toy network examples of intramodular connectivity in *PC*. Node *i* has identical intermodular connectivity in network A and B, but it has lower *PC i* in network B as it belongs to a larger blue module than network A.

PC tacitly assumes that all modules in a network are equally sized. This is, however, rarely the case in real-world networks, and nodes in small modules often have high *PC* while nodes in large modules often have low *PC* (Klimm, Borge-Holthoefer, Wessel, Kurths, & Zamora-López, 2014). This is exemplified in the two networks shown in Figure 1, where node *i* in network B, by the virtue of belonging to a larger module, has a lower PC than node *i* in network A, even though node *i* has the same intermodular connectivity in both networks. This toy network example showcases how intramodular connectivity—which is often greater in large modules—influences node participation. This issue was previously addressed by Klimm and others (2014). These authors proposed a dispersion index as well as a novel formulation of *PC*, which adjusts for the effects of intramodular connectivity to address this issue. Their formulation of *PC* involves normalizing intramodular node degrees by module size and then computing the standard deviation across the resulting normalized values. The dispersion index estimates the probability of variance between modular connections. Klimm et al. conceptualize the dispersion index as the relative inability to assign nodes to single modules. In this paper, building on the novel work of Klimm et al., we propose an alternative modification of the *PC* measure that is based on benchmarking intramodular degree using ensembles of numerically-generated null networks. This enables us to quantify the extent to which the intramodular degree of each node is greater than expected under an explicitly formulated null hypothesis. Our aim is to reduce the influence of intramodular connectivity of *PC* while retaining the underlying mathematical assumptions, and numerical range, of the original *PC* measure. To achieve this, we developed a normalized *PC* (*PC*_*norm*_) in which a node’s participation is benchmarked to an ensemble of random networks matched in node degree and connection density (Maslov & Sneppen, 2002). *PC*_*norm*_ accounts for the intramodular connectivity expected by chance, based on the spatial extent and connectivity pattern of a network’s original (i.e., nonrandomized) modules. *PC*_*norm*_, therefore, quantifies a node’s intermodular connectivity, while minimizing the influence of the same node’s intramodular connectivity. To validate *PC*_*norm*_, we used three real-world networks: (1) undirected functional MRI (fMRI) brain network data with 100 brain regions from 1,003 healthy adults (ages 22–35) participating in the Human Connectome Project (Van Essen et al., 2013); (2) a single directed out-degree network from the *C. elegans* nematode including 277 neurons (Kötter, 2004; White, Southgate, Thomson, & Brenner, 1986); and (3) a single directed out-degree flight network with 500 airports (Marcelino & Kaiser, 2012), as well as simulated networks (Guimerà & Amaral, 2005).

First, simulations were undertaken to test the hypothesis that *PC*_*norm*_ preserves the network features and numerical range of *PC*, in networks where all modules were equally sized. We then hypothesized that *PC*_*norm*_ would be less correlated with module size—here, we used module size as a proxy intramodular connectivity—than *PC* in real-world networks, across a range of resolution parameters and network density thresholds. In addition to our main hypothesis, we assessed node-specific properties of *PC*_*norm*_ to evaluate their relevance in neurobiological and air traffic systems. We also investigated whether *PC*_*norm*_ measured in fMRI networks associated with behavior more strongly than *PC*. Together, our results demonstrate a simple refinement of *PC* that retains the interpretation of *PC* in real-world networks but alleviates the effects of intramodular connectivity. Unlike the conventional *PC*, we identify London (LHR) and New York (JFK), among other airports, as high participators in the air traffic network, and demonstrate stronger associations with working memory in human brain networks. We hope *PC*_*norm*_ will lead to a more reliable estimation of nodal integration in complex networks.

## RESULTS

### Normalized Participation Coefficient (*PC*_*norm*_)

The participation coefficient (*PC*) measures whether a node interacts with only nodes from its own module or nodes from multiple modules (Guimerà & Amaral, 2005). Formally, the *PC* of node *i* is given by,

$$PC_{i}=1-\underset{m\epsilon M}{\sum }{\left(\frac{k_{i}(m)}{k_{i}}\right)}^{2},$$

where *M* is the set of network modules, *k*_*i*_(*m*) is the degree between node *i*, and all nodes in module *m* and *k*_*i*_ are the degree between node *i* and all other nodes in the entire network. A node with *PC* of zero only interacts with nodes comprising its own module, while nodes with connections uniformly distributed across all modules have a *PC* of one. As first demonstrated and comprehensively addressed elsewhere (Klimm et al., 2014), *PC* is influenced by the extent of intramodular connectivity, which may lead to inaccurate inference in networks with modules that vary in size.

To alleviate the effect of intramodular connectivity, we propose the normalized participation coefficient (*PC*_*norm*_),

$$PC_{norm_{i}}=1-\sqrt{B_{0}\underset{m\epsilon M}{\sum }{\left(\frac{k_{i}(m)-k_{i}{\left(m\right)}_{rand}}{k_{i}}\right)}^{2}.}$$

The key difference between *PC* and *PC*_*norm*_ of node *i* is subtraction of the normalization factor, *k*_*i*_(*m*)_*rand*_, from *k*_*i*_(*m*). This normalization factor denotes the median intramodular degree for node i across randomized networks generated with an established network rewiring algorithm that preserves connection density and node degree (i.e., *k*_*i*_). This also means that *k*_*i*_ is the same for original and randomized networks (Maslov & Sneppen, 2002). We found that 1,000 network randomizations were adequate to return a stable estimate of *PC*_*norm*_ (see Supporting Information Figure S1). In each randomized network, all edges were rewired five times. We used the same underlying modular network structure (*M*) for original *k*_*i*_(*m*) and random *k*_*i*_(*m*)_*rand*_. To constrain the range of *PC*_*norm*_ between 0 (low network integration) and 1 (high network integration), we add the multiplicative term *B*_0_ = 0.5, and we also calculate the square root of the difference of participation between original and randomized networks.

### PC and *PC*_*norm*_ Are Comparable in Simulated Networks with Equally Sized Modules

**Figure 2.**
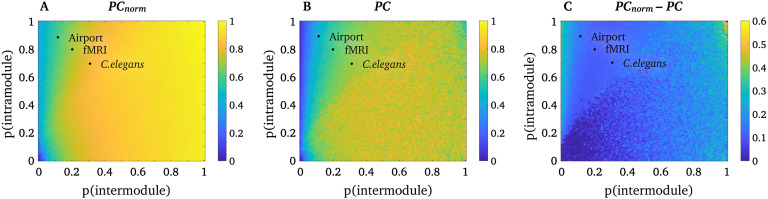
Simulations in networks with equally sized modules. Simulation results for *PC*_*norm*_and PC in networks with equally sized modules, where the heat maps show that average PC across all nodes. (A) *PC*_*norm*_across probabilities of intra- (*y*-axis) and intermodule (*x*-axis) connectivity. (B) PC across probabilities of intra- (*y*-axis) and intermodule (*x*-axis) connectivity. (C) *PC*_*norm*_minus PC across probabilities of intra- (*y*-axis) and intermodule (*x*-axis) connectivity. Black dots represent the proportion of intra- and intermodular connectivity (average of all nodes) of the three real-world networks used in this study.

First, we aimed to establish that our normalization process does not alter the conceptual basis of the *PC*. To test this assumption, we implemented the network simulation described by Guimerà and Amaral (2005) and verified that *PC* and *PC*_*norm*_ yielded comparable values in a network comprising equally sized modules. Specifically, we simulated binary networks with 100 nodes, and four modules, each containing 25 nodes. We generate networks with a predefined probability of intramodular connectivity (0–1 probability, in increments of 0.01) and intermodular connectivity (0–1 probability, in increments of 0.01), and calculated *PC*_*norm*_ (Figure 2A), *PC* (Figure 2B), and *PC*_*norm*_ minus *PC* (Figure 2C) for all possible connectivity probability values. A single network was generated for each probability value. Given that the modules were equally sized, any intramodular connectivity effects were absent by design, and thus *PC* and *PC*_*norm*_ should not markedly differ.

**Figure 3.**
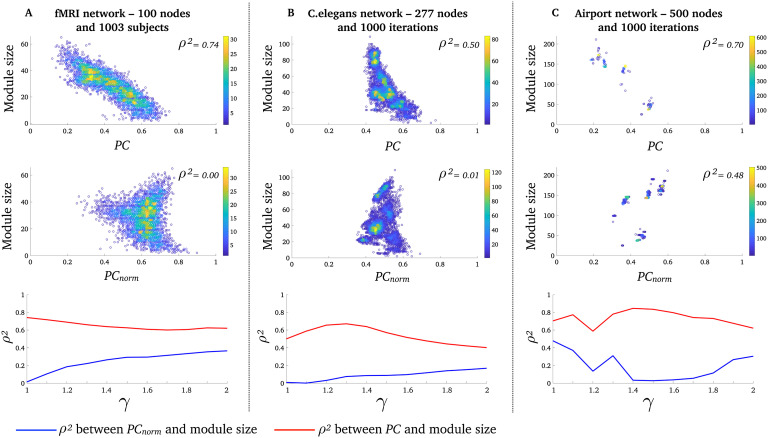
*PC*_*norm*_ is less correlated with module size than *PC*. Relationship between *PC* and module size (top row), *PC*_*norm*_ and module size (middle row), for *γ* = 1 (few and large module). Each data point is the average participation of all nodes within a module. Displayed are (A) 3,357 modules (data points) across 1,003 fMRI networks, (B), 6020 modules (data points) across 1,000 *C. elegans* networks, and (C) 3,998 modules (data points) across 1,000 airport networks. Color bars denote the density of overlapping data points. The scatter plots for the airport network appear sparser due to greater consistency across runs of the modular decomposition algorithm relative to the other networks (see Supporting Information Figure S2). The bottom row shows the mean difference between *PC*, *PC*_*norm*_, and module size for *γ* between 1 and 2 in 0.1 increments.

Figure 2A and 2B suggest that *PC*_*norm*_ and *PC* yield highly comparable estimates across a range of simulated networks with equally sized modules. More specifically, the relationship between *PC*_*norm*_ and *PC* was strong across all simulated networks (Spearman’s ρ^2^ = 0.86). This suggests that *PC*_*norm*_ remains consistent with *PC* in networks with equally sized modules, despite our algorithmic changes to node participation. Our simulation additionally shows that *PC*_*norm*_ values reside within the same range as *PC* (i.e., [0, 1]), although *PC*_*norm*_ tends to have higher values than *PC* (see Figures 2, 3, and 8).

### *PC*_*norm*_ Is Less Correlated with Module Size than *PC* in Real-World Networks

Next, we aimed to test whether *PC*_*norm*_ is less correlated with module size than *PC* and thus yields more intuitive conclusions about the importance of single nodes in three real-world networks. Using a Louvain modularity decomposition method with a spatial resolution of *γ* = 1 (Blondel, Guillaume, Lambiotte, & Lefebvre, 2008), we found that the binary and undirected fMRI human brain networks (number of nodes = 100; network density = 20%) had an average modularity *Q*-score of 0.35 ± 0.03 [SD] (*Q*-scores range between 0 and 1 where values proximate to 1 indicate that the network is highly modular, with a predominance of intramodular connections). fMRI human brain networks had an average of 3.52 ± 0.63 [SD] modules, across 1,003 healthy adults. The binary and directed (out-degree) *C. elegans* network (number of nodes = 277; network density = 5.5%) had an average modularity *Q*-score of 0.42 ± 0.01 [SD], and an average of 6.02 ± 0.55 [SD] modules, across 1,000 Louvain modularity iterations. The binary and directed (out-degree) airport network (number of nodes = 500; network density = 19%) had an average modularity *Q*-score of 0.50 ± 0.01 [SD] and an average of 4.01 ± 0.05 [SD] modules, across 1,000 Louvain modularity iterations (see Supporting Information Figure S3, for results across a range of *γ* parameters). This resulted in a total of 3,003 networks for analysis, where we for each network calculated the average *PC*_*norm*_ and *PC* across all (nonzero) nodes in each module. We also verified our results across a range of gamma resolution parameters (*γ*). We included 11 values for the *γ*-parameters (1 to 2, in increments of 0.1).

To enable statistical inference between the two squared correlation coefficients of interest (correlation value #1 = Spearman’s ρ^2^ between *PC* and module size; correlation value #2 = Spearman’s ρ^2^ between *PC*_*norm*_and module size), we employed a bootstrapping approach using 10,000 bootstrap samples, with replacement (Wilcox, 2016). Here, we aimed to test the null hypothesis of equality in the two correlation coefficients. For each sample, we computed squared correlation coefficients based on the pooled bootstrap samples of participation (*PC*_*norm*_ and *PC*) and module size, as well as its 95% confidence intervals (95% CI).

In line with our hypothesis, we found that the variance explained (ρ^2^) between the participation coefficient and module size was substantially lower for *PC*_*norm*_relative to *PC* (although *PC*_*norm*_ and *PC* were positively correlated in our three real-world networks; see Supporting Information Figure S4), where a module’s size was determined by the number of nodes it comprised. This indicates that *PC*_*norm*_ reduces the influence of intramodular connectivity compared with *PC*. We display correlation patterns between *PC*_*norm*_, *PC*, and module size, in Figure 3, and we summarize the statistical differences etween *PC*_*norm*_, *PC*, and module size in Table 1. It is worth noting there may exist nonmonotonic relationships between *PC*_*norm*_ and module size, as Spearman’s ρ is unable to detect nonmonotonic correlations.

**Table 1.** Mean ρ^2^ bootstrap difference, and 95% CI [brackets], with module size between PC and *PC*_*norm*_

	*γ* = 1	*γ* = 1.1	*γ* = 1.2	*γ* = 1.3	*γ* = 1.4	*γ* = 1.5	*γ* = 1.6	*γ* = 1.7	*γ* = 1.8	*γ* = 1.9	*γ* = 2
**fMRI network**	0.64 [0.55–0.77]	0.35 [0.29–0.44]	0.21 [0.16–0.27]	0.14 [0.12–0.20]	0.09 [0.07–0.13]	0.07 [0.05–0.09]	0.05 [0.04–0.08]	0.03 [0.03–0.05]	0.02 [0.01–0.02]	0.01 [0.01–0.02]	0.01 [0.00–0.01]
***C. elegans* network**	0.63 [0.52–0.73]	0.56 [0.49–0.70]	0.40 [0.31–0.47]	0.30 [0.21–0.32]	0.26 [0.19–0.29]	0.21 [0.18–0.28]	0.17 [0.14–0.21]	0.12 [0.10–0.16]	0.09 [0.06–0.10]	0.07 [0.05–0.08]	0.05 [0.03–0.06]
**Airport network**	0.22 [0.17–0.26]	0.40 [0.33–0.44]	0.45 [0.38–0.49]	0.47 [0.42–0.53]	0.81 [0.78–0.82]	0.81 [0.77–0.85]	0.76 [0.71–0.80]	0.69 [0.64–0.75]	0.62 [0.56–0.67]	0.41 [0.39–0.49]	0.32 [0.28–0.36]

Given that connection density—that is, the proportion of edges in a network—can markedly impact a network’s modular architecture as well as other topological properties (Zalesky et al., 2010), we also confirmed that *PC*_*norm*_ reduces the influence of intramodular connectivity compared with *PC* in fMRI networks, across multiple connection densities (Supporting Information Figure S5). From now on, we report results associated with *γ* = 1.

### Node-Wise Features of *PC*_*norm*_ in Real-World Networks

Next, we investigated the distinct inferences and conclusions that can be reached about the role of specific nodes in our proposed measure of node participation (*PC*_*norm*_). For this purpose, we subtracted average *PC* from *PC*_*norm*_across 1,003 fMRI network subjects, and 1,000 modularity runs for *C. elegans* and airport networks. In the next few paragraphs we have summarized node-wise results for fMRI, *C. elegans*, and airport networks. We have also provided a full set of node-wise results for *PC*_*norm*_ and *PC* in Supporting Information Tables S1, S2, and S3, including *z*-score differences between these two measures. A *z*-score was used to calculate the relative differences between *PC*_*norm*_ and *PC* because *PC*_*norm*_tends to yield higher values than *PC* (Figures 2 and 3). In Supporting Information Figure S6, we display the average modularity structure for fMRI, *C. elegans* and airport networks.

**Figure 4.**
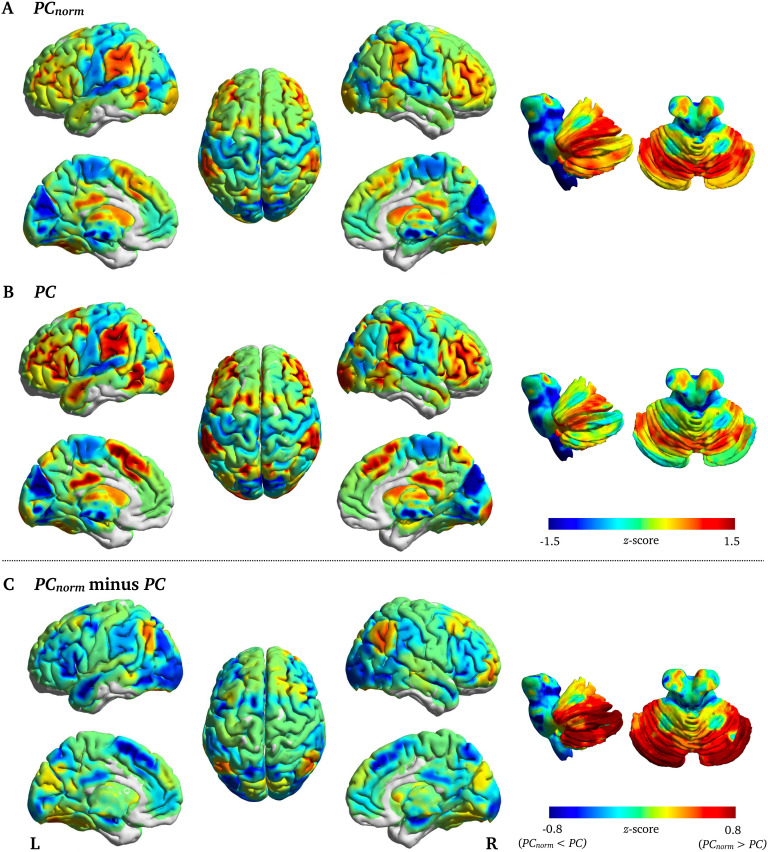
Node-wise fMRI differences between *PC*_*norm*_ and *PC*. Displayed are the subtracted group-average *PC* from *PC*_*norm*_ in fMRI networks (*z*-score). The largest difference between *PC*_*norm*_ and *PC* was observed in cerebellum—subregions lobule VI, crus I, and II—lateral parietal cortex, and insular cortex.

In the brain, the largest difference between *PC*_*norm*_ and *PC* was observed in cerebellum subregions crus I (*z*-score = 1.76), crus II (*z*-score = 1.56), and lobule VI (*z*-score = 1.42), but also in cortical areas such as inferior parietal cortex (*z*-score = 1.31) and superior frontal gyrus (*z* = 1.12) (Figure 4A), areas that are known to connect to a variety of subnetworks in the brain (van den Heuvel & Sporns, 2011) and that subserve higher order cognitive function (Shine et al., 2016). Brain regions with the highest *PC*_*norm*_ were cerebellum lobule VI (*PC*_*norm*_ = 0.73; *PC* = 0.49), cerebellum crus I (*PC*_*norm*_ = 0.72; *PC* = 0.46), inferior parietal cortex (*PC*_*norm*_ = 0.71; *PC* = 0.50), middle frontal gyrus (*PC*_*norm*_ = 0.71; *PC* = 0.52), and precuneus (*PC*_*norm*_ = 0.70; *PC* = 0.53) (Supporting Information Table S1).

**Figure 5.**
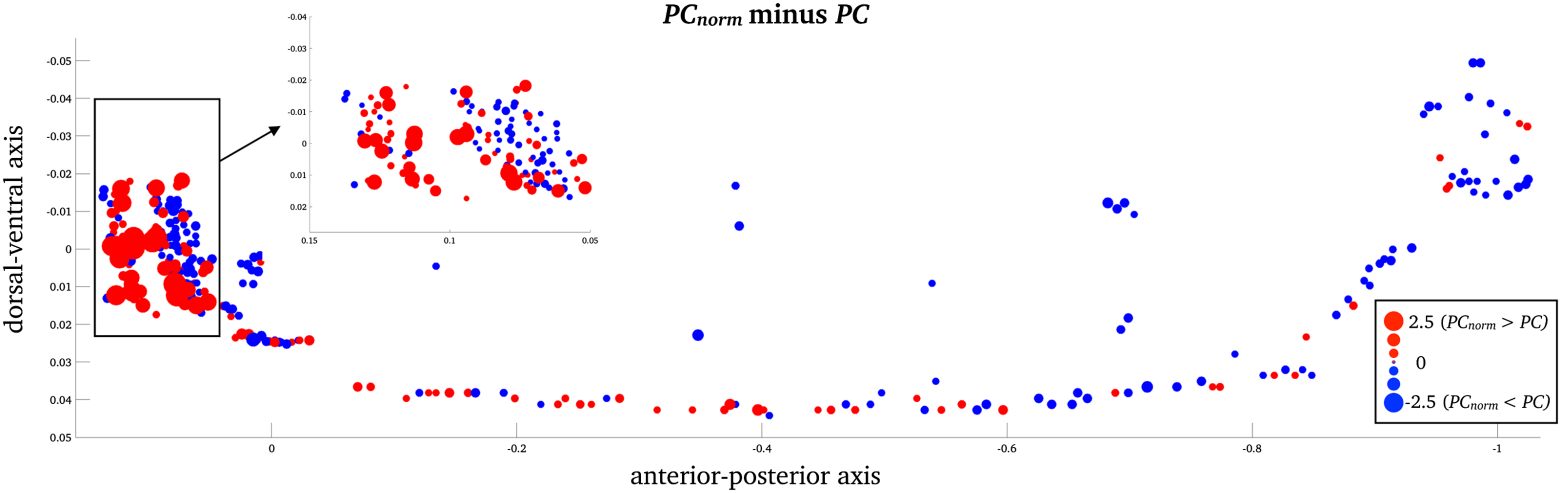
Node-wise *C. elegans* differences between *PC*_*norm*_ and *PC*. *Z*-score difference between *PC* and *PC*_*norm*_, in the *C. elegans* network (*z*-score). The largest difference between *PC*_*norm*_ and *PC*was observed in in inhibitory GABAergic neurons that belong to the largest module of the nematode.

For the *C. elegans* network, the greatest nodal differences between *PC*_*norm*_ and *PC* was observed in the largest module of the nematode encompassing inhibitory GABAergic neurons involved in head movement (RMDDL, *z*-score = 2.48; RICR, *z*-score = 2.35; RMDVL, *z*-score = 2.27; RMDDR, *z*-score = 2.26; and RMDVR, *z*-score = 2.23; Figure 5 and Supporting Information Table S2). In the *C. elegans* network, the nodes with greatest difference between *PC* and *PC*_*norm*_were not among the same nodes that had strongest node participation (*PC* and *PC*_*norm*_). The strongest *PC*_*norm*_was observed in several interneurons, also located in the head of the nematode including AVER (*PC*_*norm*_ = 0.87; *PC* = 0.75), AVAR (*PC*_*norm*_ = 0.85; *PC* = 0.75), AVAL (*PC*_*norm*_ = 0.84; *PC* = 0.75), AVBR (*PC*_*norm*_ = 0.83; *PC* = 0.74), and AVBL (*PC*_*norm*_ = 0.80; *PC* = 0.74), responsible for locomotor behavior, which is an important function for the *C. elegans* nematode. These locomotor interneurons have been found to be densely interconnected across a range of subnetworks forming a selective “rich-club” responsible for a bulk of neural signaling in the nervous system of the *C. elegans* nematode (Arnatkevičiūtė, Fulcher, Pocock, & Fornito, 2018; Bentley et al., 2016; Bertolero, Yeo, & D’Esposito, 2017; Towlson et al., 2013). See https://www.wormatlas.org/neurons/Individual%20Neurons/Neuronframeset.html for full naming, definition, and function of specific *C. elegans* neurons.

**Figure 6.**
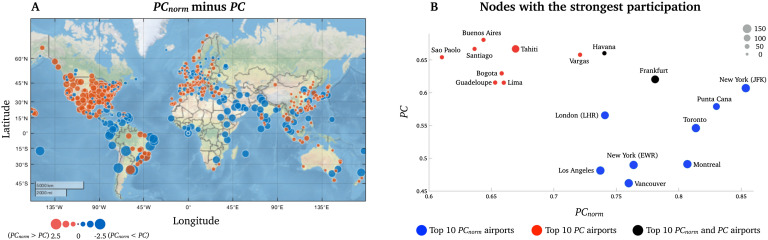
Node-wise difference between *PC*_*norm*_ and *PC* in the network of airports. (A) A world map displaying all airports included in the study. As highlighted in the manuscript, we observed the greatest difference between the two metrics in Central and South American airports (red module). (B) A visual representation of the 10 nodes with strongest *PC*_*norm*_ on the *x*-axis (blue nodes) and *PC* (red nodes) on the *y*-axis. The two black nodes were high for *PC*_*norm*_ and *PC*. Here, the size of each node is proportional to its module size (number of nodes in modules).

In the airport network, the greatest difference between *PC*_*norm*_ and *PC* was observed in South American airports (Montevideo, *z*-score = −2.26; Sao Paolo, *z*-score = −2.22; Lima, *z*-score = −2.20; Rio de Janeiro, *z*-score = −2.19; and Buenos Aires, *z*-score = −2.18; see Figure 6A and Supporting Information Table S3), which was the smallest module in this network. Notably, 8 of the 10 airports with highest PC belonged to the module encompassing Central and South America (Figure 6B, red circles). This was in contrast to the nodes with strongest *PC*_*norm*_, which included major airport hubs, including New York-JFK (*PC*_*norm*_ = 0.85; *PC* = 0.61), Punta Cana (*PC*_*norm*_ = 0.83; *PC* = 0.58), Toronto (*PC*_*norm*_ = 0.81; *PC* = 0.55), Montreal (*PC*_*norm*_ = 0.81; *PC* = 0.49), and Frankfurt (*PC*_*norm*_ = 0.78; *PC* = 0.62) (see blue circles in Figure 6). These airports are known to have many intercontinental flights. This node-wise analysis suggests that *PC* overestimates the integrative nature of nodes within relatively small modules and that *PC*_*norm*_ can alleviate *PC*’s influence of intramodular connectivity, thereby enabling clearer and more intuitive conclusions to be drawn about the intermodular connectivity of nodes.

### *PC*_*norm*_ Is More Strongly Correlated with Working Memory Performance than *PC*

We next aimed to test whether interindividual variation in *PC*_*norm*_measured in brain networks would associate more strongly with behavioral measures than PC (Barch et al., 2013).

**Figure 7.**
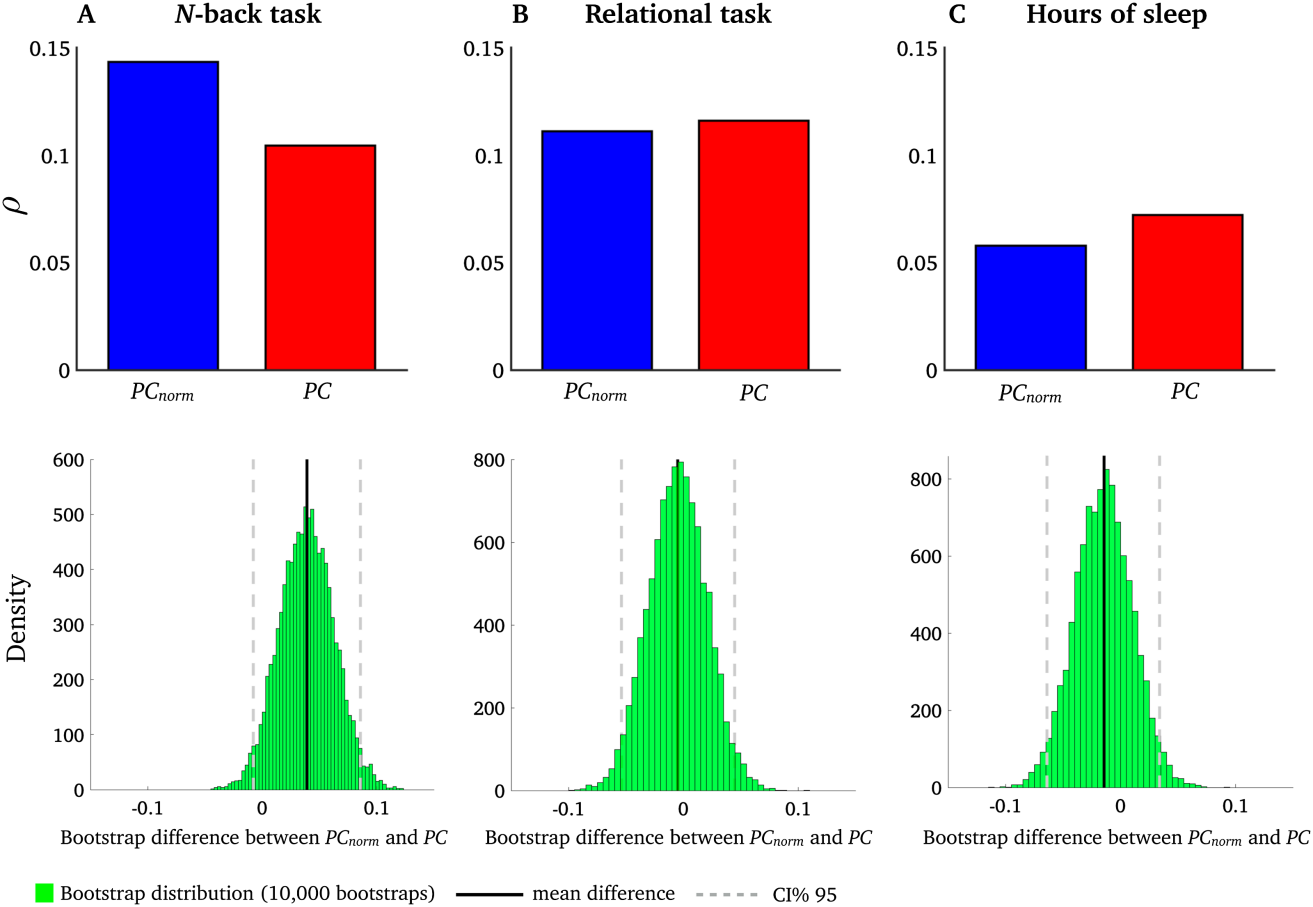
Correlation between *PC*_*norm*_, *PC*, and behavior. Interindividual variation in *PC*_*norm*_ (blue) and *PC* (red) was associated with three behavioral measures: (A) *N*-back task, (B) sleep index, and (C) relational task. Spearman’s ρ between *N*-back task and *PC*_*norm*_ was 0.14 (*df* = 1,001, *p* < 0.00001); Spearman’s ρ between *N*-back task and *PC* was 0.11 (*df* = 1,001, *p* < 0.001). Bootstrap difference between *PC*_*norm*_ and *PC* for *N*-back task was 0.04 [95% *CI* = −0.01 to 0.08]. Spearman’s ρ between relational task and *PC*_*norm*_ was 0.11(*df* = 1,001, *p* < 0.001); Spearman’s ρ between relational task and PC was 0.12(*df* = 1,001, *p* < 0.0001). Bootstrap difference between *PC*_*norm*_ and *PC* for relational task was 0.01 [95% *CI* = −0.05 to 0.05]. Spearman’s ρ between sleep and *PC*_*norm*_ was 0.06 (*df* = 1,001, *p* = 0.07); Spearman’s ρ between sleep and *PC* was 0.07 (*df* = 1,001, *p* = 0.04). Bootstrap difference between *PC*_*norm*_ and *PC* for sleep was 0.01 [95% *CI* = −0.05 to 0.06].

We conducted an analysis informed by our previous study where we showed that intermodular network switching was related to the following behavioral domains (Pedersen et al., 2018): (1) *N*-back task, calculated as the average of 0- and 2-back task, important for working memory performance; (2) a relational task central for planning and reasoning; and (3) a sleep index averaging hours of sleep the month before the fMRI scan. Each subject had a single behavioral score for each of the three behavioral domains. We therefore averaged *PC*_*norm*_ across all nodes in the network to yield a single *PC*_*norm*_value for each subject. We show that, using the same bootstrapping procedure outlined previously, mean *PC*_*norm*_ associated with functional brain networks was correlated more strongly with the *N*-back task compared with *PC*, but not the sleep index and relational task (Figure 7). This suggests that working memory performance relates to the extent of intermodular connectivity, rather than the size, of modules.

### *PC*_*norm*_, *PC*, and Within-Module Degree *Z*-Score in Real-World Networks

**Figure 8.**
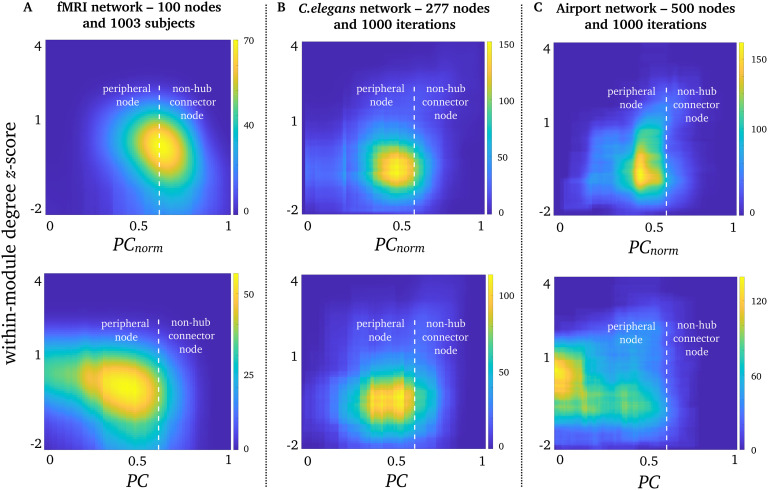
Participation and within-module degree *z*-score. Joint histograms containing the number of simultaneous occurrences between *PC*_*norm*_ (top row), *PC* (bottom row), and within-module degree *z*-score in (A) fMRI networks, (B) *C. elegans* networks, and (C) airport networks. Within-module degree *z*-score is shown on the vertical axis, and *PC* and *PC*_*norm*_ are shown on the horizontal axis. In accordance with Guimerà and Amaral (2005), *PC* of 0.62 was used to distinguish between peripheral nodes (*PC* < 0.62) and non–hub connector nodes (*PC* > 0.62), and a within-module degree *z*-score < 2.2.

*PC* is often interpreted alongside within-module degree *z*-score, a metric that quantifies the normalized degree of intramodular nodes. First of all, we observed almost no correlation between within-module degree *z*-score and module size (Spearman’s ρ^2^ = 0.01, for *γ* of 1). We next projected corresponding values between *PC* and within-module degree *z*-score in a joint histogram which categorizes the intra- and intermodular statuses of nodes (see Figure 8). Similarly to previous work, most network nodes are peripheral nodes classified as nodes that mainly connect to a single module (Guimerà et al., 2005). Note that this definition of peripheral nodes is unrelated to core-periphery networks previously outlined by Csermely, London, and Uzzi (2013). However, our joint histogram analysis showed that *PC*_*norm*_ detected more non–hub connector nodes than *PC* in fMRI networks (Figure 8A). Non–hub connector nodes are classified as nodes that connect to a variety of modules. Airport networks also showed increased *PC*_*norm*_ compared with *PC*, but remained as peripheral nodes (Figure 8B). On the other hand, *PC* and *PC*_*norm*_ yielded comparable inference about the *C. elegans* network, which primarily comprised peripheral nodes (see Figure 8C). This suggests that *PC*_*norm*_may alter the topological structure, and interpretation, of networks especially in modules of varying sizes.

## DISCUSSION

We proposed a normalized participation coefficient (*PC*_*norm*_) to alleviate the effects of intramodular connectivity in the established formulation of *PC* developed by Guimerà and Amaral (2005). Using brain, *C. elegans*, airport, and simulated networks, we demonstrated that our proposed measure of participation (1) alleviates influence of intramodular connectivity in networks with varying module sizes, (2) preserves the conceptual and mathematical properties of the classic formulation of *PC*, and (3) yields new insights into the role and function of nodes in the context of their participation across modules, thereby enabling clearer and more intuitive conclusions to be drawn about the role of nodes. Our work should be conceptualized as an extension to Guimerà and Amaral’s classic formulation of *PC*, rather than as a new measure to include in the network scientist’s toolbox. We believe *PC*_*norm*_ may be useful, particularly for networks with significant variation in module size. These are the networks for which the intramodular connectivity influences the classic *PC*.

It is important to clarify and highlight the main reason why we want to reduce the influence of intramodular connectivity in *PC*, namely network modularity. Modularity decomposition methods are designed to search for a collection of nodes that have stronger and more widespread interconnectedness than we expect by chance (Blondel et al., 2008). Then, a sizable collection of interconnected nodes forming a large module will have low *PC* values without necessarily reflecting the node’s interconnectedness with other modules. An example of this is the case of airport networks. We observed maximal *PC* in a subset of airports located in the smallest module comprising Central and South America that consequently led to high *PC* values (Figure 6). After reducing the influence of intramodular connectivity, *PC*_*norm*_ suggested that the airports with strongest intermodular connectivity were located in metropolitan cities such as New York, London, and Paris, where over one-third of flights were cross-continental. It is also important to note that *PC*_*norm*_, like *PC*, appears independent from node degree. This is evident as the two airports with highest *PC*_*norm*_ had highly divergent node degree: (1) New York had a total of 218 connections (127 intramodular connections), and (2) Punta Cana in the Dominican Republic had only 52 connections (34 intramodular connections). Although Punta Cana is not a densely connected airport, it belongs to the large module encompassing North America. This airport has a substantial number of cross-continental flights, mainly to South America, again demonstrating how *PC* may underestimate the integrative nature of nodes within large, and highly intraconnected, modules. It is important for future studies to establish whether these distinct differences between *PC*_*norm*_ and *PC* are generalizable to a range of real-world networks.

An example of the utility of *PC*_*norm*_ in real-world networks was seen in fMRI brain networks, where we observed increased intersubject correlation with the *N*-back task compared with *PC* (Figure 7A). This task is important for working memory performance (Kirchner, 1958), a higher order cognitive process that engages a distributed brain network comprising symmetric and bilateral frontal and parietal cortices (LaBar, Gitelman, Parrish, & Mesulam, 1999; Meyer, Spunt, Berkman, Taylor, & Lieberman, 2012). We found that *PC*_*norm*_ was relatively high in these regions compared with other areas of the cortex, but this was not as prominent as PC. Several reports also suggest that *PC* is correlated with working memory performance (Bertolero et al., 2018; Cohen & D’Esposito, 2016; Shine et al., 2016; Stanley, Dagenbach, Lyday, Burdette, & Laurienti, 2014). For example, Shine et al. (2016) reported increased *PC* in fronto-parietal networks during the *N*-back task compared with the resting state. This suggests that the human brain may allocate more intermodular connectivity to meet demands of this cognitively strenuous task. Other brain regions that expressed relatively high values of *PC*_*norm*_ were located in the cerebellum, mainly subregion Crus I and Lobule VI (see Figure 4A). These cerebellar subregions are also involved in working memory (Desmond & Fiez, 1998; Marvel & Desmond, 2010; Schmahmann & Caplan, 2006). They display connectivity with frontal and parietal cortices (Allen et al., 2005; Bernard et al., 2012) and have preferentially expanded over recent evolutionary time (Shine & Shine, 2014).

Unlike *PC*, nodes that only have intramodular connections will not have a *PC*_*norm*_ value of zero. This is because our randomization approach always returns a “random” number to subtract from the original *PC* algorithm. We found that, in real-world networks, *PC*_*norm*_ tends to be low when *PC* is zero (Supporting Information Figure S7). However, our simulations showed a difference between *PC*_*norm*_ and *PC* in strongly connected nodes in a lattice network (see upper left corner of Figure 2C). This network configuration was not observed in our three real-world networks (see black dots in Figure 2).

Regressing module size from the original *PC* is an alternative to *PC*_*norm*_. This is done by constructing a linear regression model where *PC* of nodes constitute the dependent variable, and the module size associated with each node is the independent variable. Subtracting the fit of the regression model from the original *PC* results in a *residual PC* score. Within this regression framework, a positive residual *PC* score is observed for nodes with participation that is higher than expected because of module size effects alone, whereas negative residual *PC* is observed for nodes with participation that is lower than expected because of module size. Residual PC close to zero indicates that nodes are strongly correlated with module size. Another alternative to *PC*_*norm*_ is to exclude intramodular connectivity altogether. This can be achieved by quantifying a node’s between-module degree, that is, the number of connections from node *i* to all other nodes that does not belong to node *i*’s module. These additional measures may be useful but require further investigation.

We provide code to calculate *PC*_*norm*_, in addition to residual *PC* and between-module degree, as described in the previous paragraph, available on GitHub (https://github.com/omidvarnia/Dynamic_brain_connectivity_analysis). The current version of *PC*_*norm*_ works for undirected/directed and weighted/binary networks. A limitation of *PC*_*norm*_ is the computational time needed for network randomizations, which can be particularly time consuming for large networks (i.e., > 1,000 nodes). To circumvent this issue, we added a parallel computing option in our code that allows for faster computation time, ensuring this process is tractable for all but the largest networks. Nevertheless, as network randomization procedures are becoming increasingly popular, it is imperative to continue optimizing computational capabilities when randomizing edges in large and complex networks.

## METHODS

### Modular Decomposition of Networks

Before estimating *PC* and *PC*_*norm*_ (defined in the Results section), we parsed fMRI, *C. elegans*, and airport networks into modules by using a Louvain modularity optimization procedure (Blondel et al., 2008) written as:

$$[Q,M]=\frac{1}{2m}\underset{ij}{\sum }\left[A_{ij}-\gamma \frac{k_{i}k_{j}}{2m}\right]\delta \left(c_{i},c_{j}\right),$$

where *A*_*ij*_ represents the binary connectivity edge between nodes *i* and *j*. Louvain modularity optimization procedure finds nodes with greater intramodular connectivity that is expected by chance using a null model where *k*_*i*_*k*_*j*_ (summation of all edge weights attached to nodes *i* and *j*) is divided by 2*m* (*m* being the summation of all edge weights); *c*_*i*_ and *c*_*j*_ are the modules associated with node *i* and *j*, and the module vector output is *M*; and *γ* controls the spatial resolution of modules. A low *γ* (*γ* < 1.5) results in a few large modules, whereas a high *γ* (*γ* > 1.5) returns numerous small modules (Bassett et al., 2013). We validated our findings across a variety of *γ* parameters between 1 and 2 in 0.1 increments, which is a relevant range of gamma values in our networks. The Louvain clustering method also contains heuristics that may cause run-to-run variability due to the degeneracy issue in complex networks (Good, de Montjoye, & Clauset, 2010). This provided us with a good opportunity to assess *PC* across a range of modular structures in the *C. elegans* and airport network where we only had a single network available for analysis. In these two networks, we obtained 1,000 different modular outputs. In fMRI networks we computed a single modular output of each of the 1,003 subjects by using a consensus clustering approach with 1,000 iterations (Lancichinetti & Fortunato, 2012). For each modularity run in *C. elegans* and airport networks, and fMRI network subject, we obtained a 1 × *N* vector where *N* is total number of nodes (i.e., 100 nodes for fMRI networks, 277 nodes for the *C. elegans* neuronal system, and 500 nodes for the airports network) comprising *m*-numbers of modules (*M* = all modules).

### Within-Module Degree *Z*-Score

To compare *PC* and *PC*_*norm*_ with intramodular connectivity, we computed within-module degree *z*-score, that is, the normalized connectivity of a node within its own module (Guimerà & Amaral, 2005). It is given by:

$$z_{i}=\frac{k_{i}\left(m_{i}\right)-\overset{-}{k}(m_{i})}{\sigma^{k(m_{i})}},$$

where *m*_*i*_ denotes the module containing *i*, *k*_*i*_(*m*) is the overall degree of node *i* in module *m* (where node *i* is located), $\overset{-}{k}$(*m*_*i*_) is the mean degree of all nodes in module *m*, and *σ*^*k*(*m*_*i*_)^ is the standard deviation of all nodes in module *m*.

### Dataset 1: fMRI Network

We used 57.6 min (4,800 time points × 0.72-s repetition time) of resting-state fMRI data from 1,003 healthy adults between ages of 22 and 35 years, from the Human Connectome Project (Van Essen et al., 2013), that we filtered between frequencies of 0.01 and 0.1 Hz. The fMRI data was distortion, motion, and field bias–corrected and normalized into a common Montreal Neurological Institute space. An independent components analysis was conducted to parcellate the brain into 100 regions of interests (55 subcortical and 45 cortical brain regions). At the individual level, we generated an undirected binary brain network with 100 nodes by using pair-wise Pearson correlation analysis of subject-specific fMRI time series over 100 brain regions. For our main analysis we thresholded networks at a sparsity level 20% (990 edges). We also derived networks at additional sparsity levels of 10% (495 edges), 30% (1,485 edges), and 40% (1,980 edges; Supporting Information Figure S5).

### Dataset 2: *C. elegans* Network

We analyzed two-dimensional spatial representations of the global neuronal network (277 neurons, or nodes, and 2,105 connections, or edges) of the nematode *C. elegans*, previously identified by electron microscope reconstructions (White et al., 1986). This network is directed and binary, that is, it contains both incoming and outgoing neural connections between brain areas of the *C. elegans*. In this study we only used outgoing connections.

### Dataset 3: Airport Network

Airport network was constructed based on flights between the 500 busiest airports (nodes) in the world. Edges were based on the total passenger volume between airports. In this network there were a total of 24,009 flights (edges). The existence of flight connections between airports is based on flights within one year from 1 July 2007 to 30 June 2008 (Marcelino & Kaiser, 2012). An edge means that there is at least one flight between two airports. This network is directed and binary, meaning it contains incoming and outgoing flight connections between airports. In this study we only used outgoing connections.

## SUPPORTING INFORMATION

Supporting Information for this article is available at https://doi.org/10.1162/netn_a_00127.

## AUTHOR CONTRIBUTIONS

Mangor Pedersen: Conceptualization; Data curation; Formal analysis; Investigation; Methodology; Software; Validation; Visualization; Writing - Original Draft. Amir Omidvarnia: Conceptualization; Formal analysis; Methodology; Validation; Writing - Review & Editing. James M Shine: Conceptualization; Formal analysis; Investigation; Methodology; Visualization; Writing - Review & Editing. Graeme D. Jackson: Conceptualization; Funding acquisition; Investigation; Resources; Supervision; Validation; Writing - Review & Editing. Andrew Zalesky: Conceptualization; Investigation; Methodology; Supervision; Validation; Visualization; Writing - Review & Editing.

## FUNDING INFORMATION

Graeme D. Jackson, National Health and Medical Research Council (http://dx.doi.org/10.13039/501100000925). Andrew Zalesky, National Health and Medical Research Council Senior Research Fellowship B (APP1136649).

## TECHNICAL TERMS

Participation coefficient (*PC*): Estimates how well a node within a given module is connected to other brain-wide modules. It is a measure of intermodular diversity.
fMRI: An imaging technique that captures hemodynamic interactions in the brain.
*C. elegans*: A nematode with 302 neurons that is approximately 1-mm long.
Gamma resolution parameter: A parameter that influences the size and extent of network modules.
Network modules: A collection of network nodes with greater interconnectivity than expected by chance.
Normalized participation coefficient (*PC*_*norm*_): Similar to original participation coefficient, but it reduces the influence of intramodular connectivity.
Within-module degree *z*-score: Normalized degree centrality, i.e., number of links within a module.
