# Supplementary material for: Reducing the influence of intramodular connectivity in participation coefficient
Source: Netw Neurosci. 2020 Apr 1;4(2):416–31. doi: 10.1162/netn_a_00127 (PMC7286311; doi:10.1162/netn_a_00127)
Supplement: Supplementary file 1 [file netn-04-416-s001.pdf]

Pedersen, M., Omidvarnia, A., Shine, J. M., Jackson, G. D., & Zalesky, A. (2020). Supporting information for "Reducing the influence of intra-modular connectivity in participation coefficient." *Network Neuroscience*, *4*(2), 416–431.
https://doi.org/10.1162/netn_a_00127

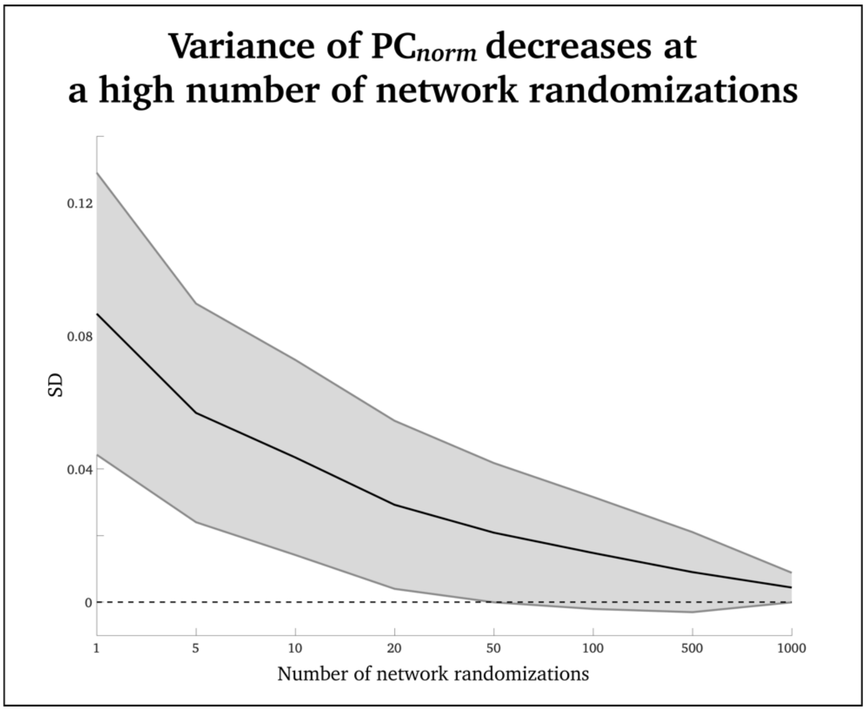

Supplementary figure 1: We wanted to test how many network randomizations are needed to return a stable estimate of PC_{norm}. For this purpose, we used a single randomly selected fMRI networks and derived PC_{norm} from 8 different network randomization parameters (1, 5, 10, 20, 50, 100, 500 and 1000 randomizations). For each of these 8 randomization parameters, we computed 1000 networks. Then the SD across the 1000 networks was computed, for each randomization parameter. We observed less variance across 1000 network iterations at a high number of network randomizations (up to 1000 network randomizations). Nevertheless, variance was also relatively low when computing 100 network randomizations, which may be more feasible in large networks due to the computational burden of network randomizations.

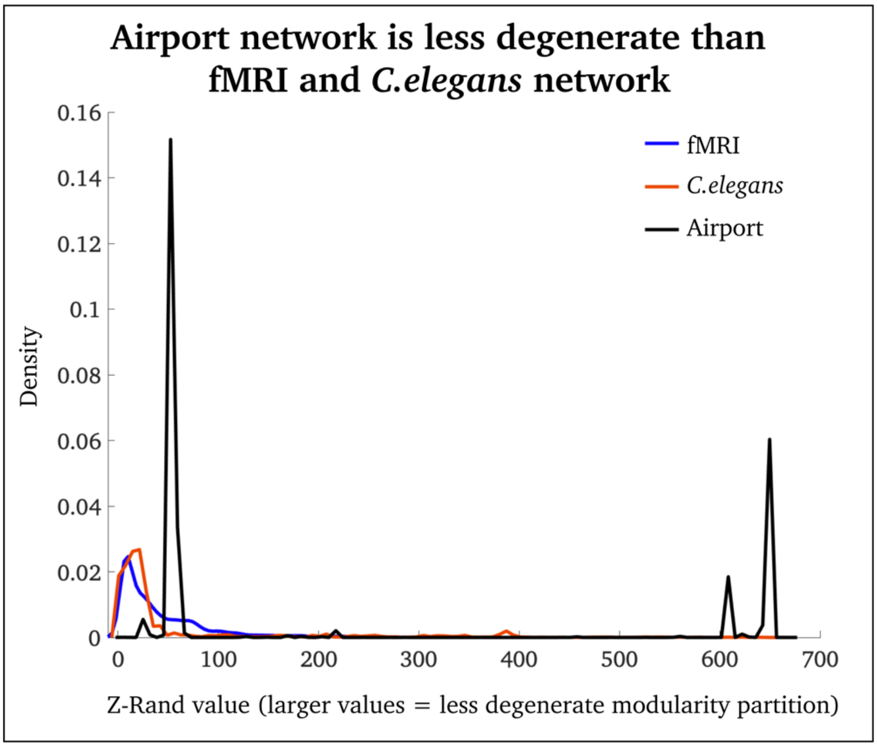

Supplementary figure 2: In this figure we show that the airport network (black distribution) is less degenerate than fMRI (blue distribution) and *C.elegans* (red distribution) networks, where larger *Z*-rand values means that the network is less degenerate. Degeneracy refers to the stability of network outputs modularity iterations (fewer stable outputs = more degeneracy). For a more thorough introduction to the *Z*-rand metric, refer to Traud et al, SIAM Review 53, 526 (2011).

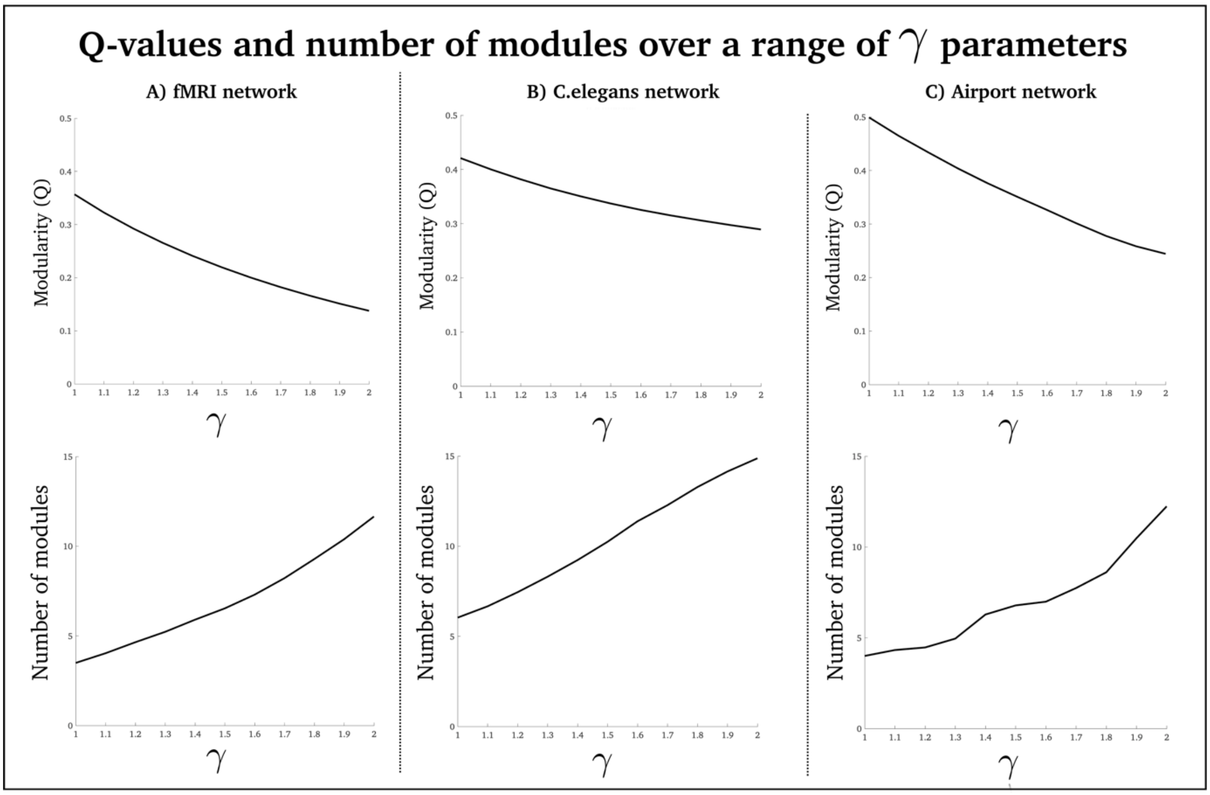

Supplementary figure 3: We computed modularity (Q-value) the average number of modules across a range of gamma parameters (1 to 2, in 0.1 increments). In this figure we present the median value across subjects (fMRI – A) and modularity runs (*C.elegans* – B; airport – C). We observed that modularity decreased, and number of modules increased, as gamma increased, across all three network types. This suggest that the networks may lose their inherent modular structure at high gamma values.

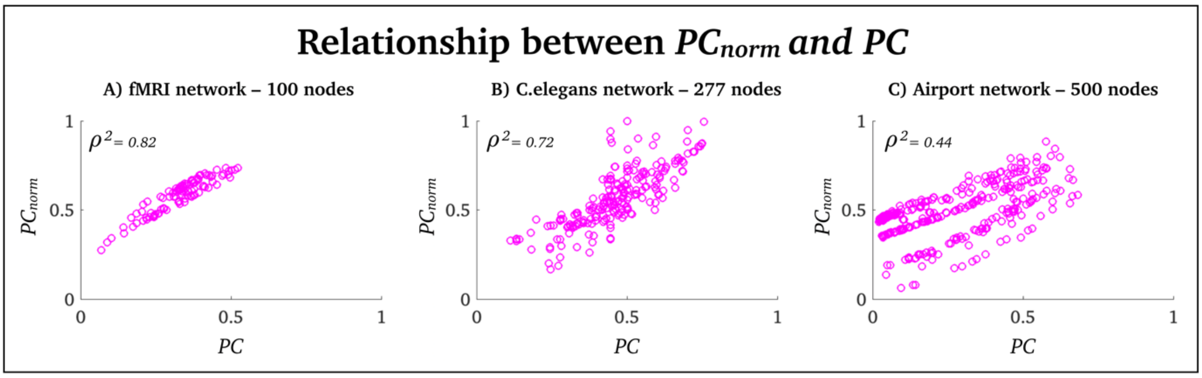

Supplementary figure 4: We generated scatterplots between all PC and PC_{norm} nodes in our three real-world networks. In the scatterplots seen below, each pink dot denote a single node, averaged over 1003 subjects (section A – fMRI network) and 1000 modularity iterations (section B – *C.elegans* network; section C – airport network). These scatterplots demonstrate that there is a positive correlation between PC and PC_{norm}. This finding supports our simulation results (Figure 2 in manuscript), suggesting that PC and PC_{norm} are measuring similar topological features in the network.

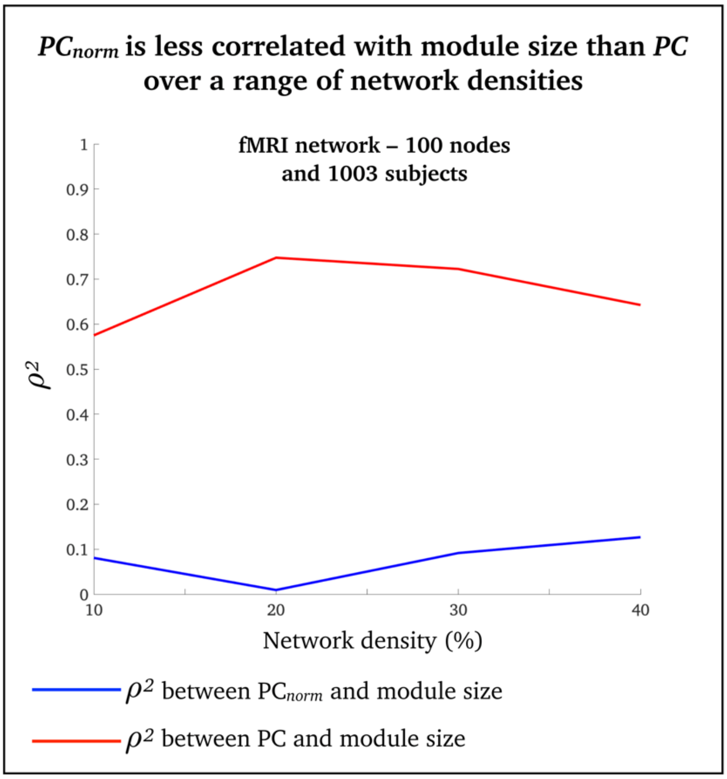

Supplementary figure 5: $\mathrm{PC}_{\mathrm{norm}}$ is less correlated with module size than PC over a range of network densities. Connection density can markedly impact a network's modular architecture as well as other topological properties. Hence, we aimed to test whether variance explained between PC, PC_{norm} and module size differed across network densities. To this end, we thresholded each of the 1,003 brain networks to achieve connection densities of 10%, 20%, 30% and 40%. We were not able to use *C.elegans* and airport networks for this analysis as these network datasets were pre-thresholded and binarized before public release. We observed lower correlations between PC_{norm} and module size (Spearman's ρ^2 ranged between 0.00 and 0.19) compared to PC and module size (Spearman's ρ^2 ranged between 0.56 and 0.75), across all connection density thresholds. The bootstrapped ρ^2 difference (10,000 bootstraps) between PC_{norm} and PC ranged between 0.47 [95% CI = 0.44 – 0.50] and 0.75 [95% CI = 0.73 – 0.76], across all connection density thresholds.

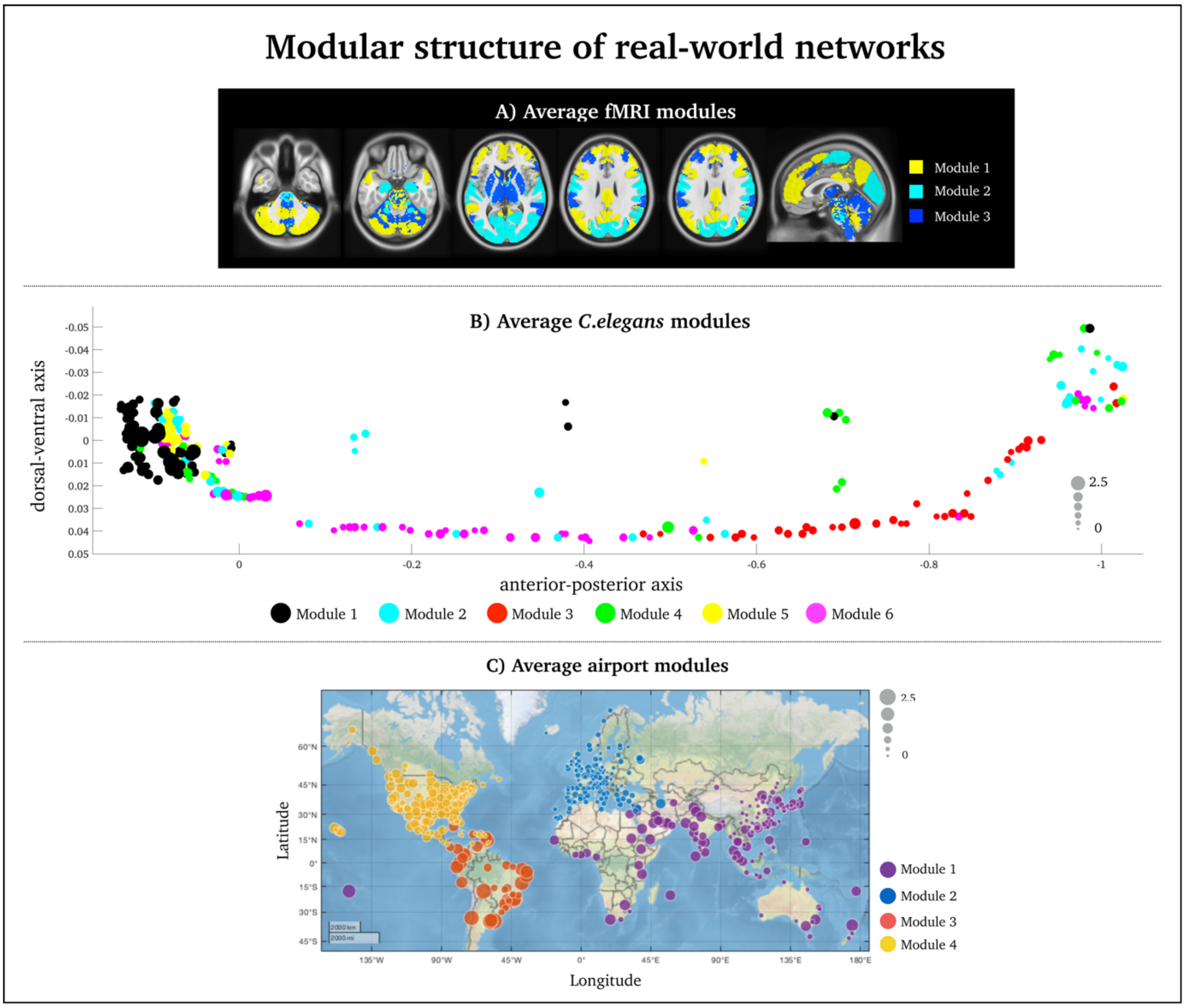

Supplementary figure 6: Modularity structure in real-world networks (γ = 1). (A) Group-level fMRI modularity map derived from a consensus clustering algorithm. Consensus clustering algorithms derive a single fMRI module (1 x 100 node vector) that best describes the modular information from all subjects and nodes (1003 subjects x 100 node array). (B) The colors denote distinct modules derived from a consensus clustering algorithm across 1000 modularity iterations. The black module displayed on the left of the figure comprises inhibitory GABAergic neurons that belong to the largest module of the nematode encompassing RMDDL, RICR, RMDVL, RMDDR and RMDVR neurons. (C) Colors denote modules derived from a consensus clustering algorithm across 1000 Louvain modularity iterations.

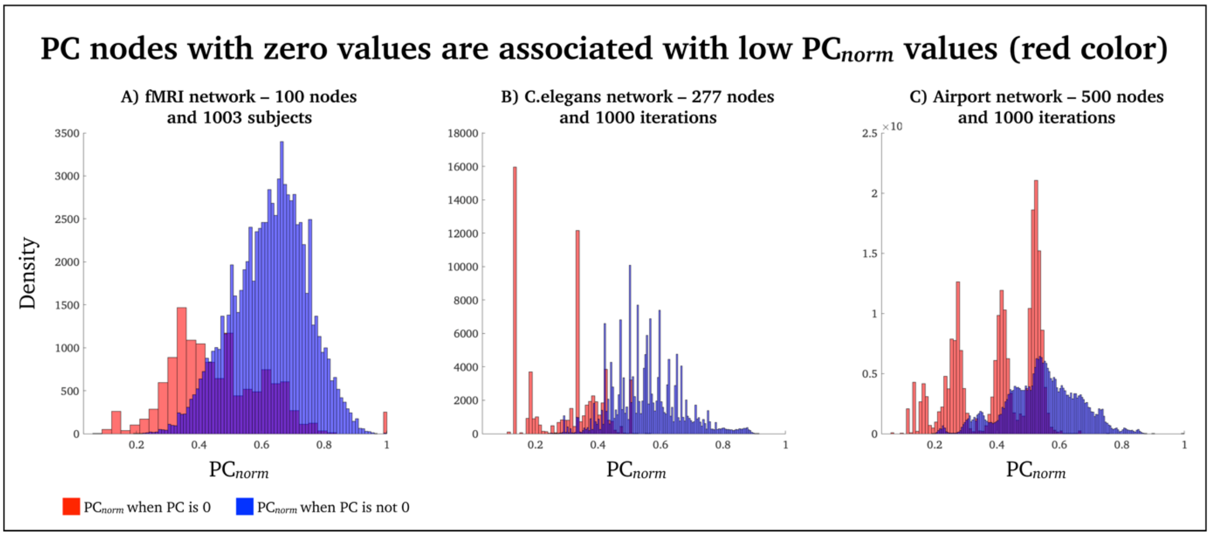

Supplementary figure 7: As we discussed in the main manuscript, nodes that only have intra-modular connections will not have a PC_{norm}value of zero (unlike PC). This is because our randomization approach always generates a 'random' number to subtract from the original PC algorithm. The red distributions in this figure reflects all PC_{norm}nodes that corresponds to PC nodes with values of zero. It is evident that PC_{norm} tends to be low when PC is zero (red distribution) compared to when PC is not zeros (blue distribution), across A) fMRI, B) *C.elegans* and C) airport networks.

***Supplementary table 1: fMRI* network:** Full set of nodal results for PC_{norm} and PC, and z-score differences between PC_{norm} and PC. Note we used a z-score since PC_{norm} tends to yield higher values than PC -- see Fig. 2 and 3). The last column displays the module community affiliation for each node.

	**fMRI node (AAL definition)**	**PC_{norm}**	**PC**	**Z-score difference**	**Module**
1	Cerebelum_6_L	0.725951212	0.489386403	0.567616364	1
2	Cerebelum_Crus1_BIL	0.719282621	0.456551875	1.093689928	2
3	Parietal_Inf_R	0.708799866	0.499882327	0.011760156	1
4	Middle Frontal Gyrus_BIL	0.707511318	0.521348924	-0.445738491	1
5	Precuneus_BIL	0.701201163	0.53041552	-0.75489244	3
6	Cerebelum_Crus1_R	0.699871394	0.428270238	1.272032053	2
7	Insula_R	0.696412289	0.520064242	-0.643058737	2
8	Parietal_Inf_R	0.690456671	0.482024811	0.001995438	1
9	Cerebelum_Crus1_R	0.689182966	0.418089464	1.261825526	2
10	Cerebelum_Crus2_BIL	0.687837526	0.402389567	1.550425938	2
11	Cerebelum_Crus2_L	0.685835304	0.407177106	1.413915897	1
12	Cerebelum_Crus1_L	0.683617476	0.445404201	0.600759243	2
13	Caudate_dorsal_BIL	0.683271034	0.460794317	0.284371278	1
14	Cerebelum_6_BIL	0.68268362	0.437801579	0.734836656	2
15	Cerebelum_8_R	0.680244395	0.41485296	1.147183857	2
16	Cerebelum_7b_R	0.679796826	0.401726195	1.402102678	1
17	Precuneus_BIL	0.679376428	0.483770649	-0.255876548	1
18	Parietal_Sup_L	0.67933796	0.514261427	-0.869675716	1
19	Thalamus_L	0.678833896	0.443459874	0.543675239	1
20	Cerebelum_Crus1_R	0.672781211	0.392688835	1.442750444	1
21	Insula_L	0.668925572	0.509384743	-0.980972612	1
22	Cerebelum_8_L	0.663320703	0.412712444	0.84996391	2
23	Cerebelum_Crus2_L	0.660374916	0.379925207	1.449934721	1
24	Cerebelum_6_L	0.658623439	0.379776311	1.417714377	2
25	Cerebelum_7b_L	0.657134246	0.396904637	1.043403875	1
26	Vermis_8	0.65628065	0.414777894	0.666895176	3
27	Vermis_4	0.648899941	0.415845938	0.497030613	1
28	Cerebelum_6_L	0.646939909	0.373842709	1.302110443	1
29	Cerebelum_6_R	0.643372756	0.362136018	1.465758171	2
30	Cerebelum_6_R	0.643307166	0.380369987	1.097840336	1
31	Inferior Parietal Lobule_R	0.642520319	0.434530476	-0.00689144	3
32	Cerebelum_Crus2_R	0.642511289	0.356204154	1.567699938	1
33	Middle Frontal Gyrus_R	0.642210271	0.472533118	-0.777178945	2
34	Cerebelum_8_R	0.641895261	0.379818496	1.080541471	1
35	Occipital_Mid_BIL	0.640190742	0.464514998	-0.656575566	1
36	Cerebelum_Crus1_L	0.638851414	0.349244781	1.634037279	2
37	Cerebelum_8_L	0.637358197	0.433325552	-0.086452018	2
38	Cerebelum_6_L	0.635117564	0.381186409	0.91677167	1
39	Cerebelum_Crus1_R	0.633504629	0.352896195	1.453125934	1
40	Cerebelum_7b_R	0.630269707	0.351818813	1.409747967	2
41	Caudate_ventral_BIL	0.629419965	0.39964153	0.431174388	1
42	Superior Frontal Gyrus_R	0.626212259	0.361951022	1.124460893	2
43	Angular_L	0.624061779	0.393933949	0.438199068	1
44	Occipital_Mid_R	0.623814323	0.470296181	-1.10206044	2
45	Occipital_Sup_R	0.623754267	0.458167468	-0.859416657	1
46	Middle Temporal Gyrus_BIL	0.619242895	0.442314584	-0.631392361	2
47	Frontal_Mid_Orb_L	0.617835871	0.398686201	0.217480052	1
48	Cerebelum_Crus1_L	0.617033927	0.313827139	1.907472245	2
49	Cerebelum_6_R	0.617027459	0.391339691	0.348930366	2
50	Cerebelum_8_L	0.616260913	0.404553054	0.067860315	2
51	Putamen_ant_BIL	0.615960006	0.397418914	0.205244413	2
52	Putamen_post_BIL	0.615396757	0.384433481	0.454995947	2
53	Cerebelum_6_L	0.612024349	0.372582107	0.62546797	2
54	Putamen_mid_BIL	0.60662399	0.404821032	-0.131280519	2
55	Cerebelum_8_R	0.606290296	0.402041408	-0.082104406	2
56	Frontal_Sup_Medial_R	0.606018819	0.360412077	0.749406974	2
57	Angular_L	0.601316248	0.342745353	1.010054962	2
58	Thalamus_BIL	0.600835109	0.41521247	-0.456590426	3
59	Frontal_Inf_Orb_L	0.598774969	0.392050394	-0.032330017	2
60	Cerebelum_Crus1_R	0.596773279	0.300963207	1.758759188	2
61	Parietal_Inf_R	0.596010994	0.337935742	1.000089929	2
62	Parietal_Inf_L	0.593605293	0.450380407	-1.309009282	1
63	Medulla	0.589977118	0.383980779	-0.046971401	3
64	Brainstem_L	0.586985607	0.36197052	0.33540592	2
65	Calcarine_L	0.581827878	0.435031595	-1.237205304	3
66	Temporal_Mid_L	0.581701092	0.40689825	-0.6741255	2
67	Occipital_Mid_L	0.578437473	0.415595828	-0.914608752	3
68	Parietal_Sup_L	0.570980573	0.389978683	-0.549491883	2
69	Putamen_L	0.56969866	0.381170914	-0.398182425	3

70	Substania Nigra_L	0.566440702	0.391396332	-0.669269517	1
71	Parahippocampa Gyrus_BIL	0.565564426	0.419427381	-1.250459483	2
72	Hippocampus_R	0.563331005	0.373402657	-0.370022936	3
73	Substania Nigra_R	0.56141693	0.367824025	-0.29634596	2
74	Pons	0.560676466	0.411730107	-1.193977432	2
75	Brainstem	0.560323184	0.398835372	-0.941827964	1
76	Precuneus_L	0.556986968	0.369647092	-0.42206488	3
77	Right Brainstem	0.555267266	0.412840965	-1.325065044	1
78	Occipital_Mid_L	0.555239553	0.392892792	-0.924558558	2
79	Occipital_Inf_L	0.545434379	0.381949455	-0.90167545	3
80	Midbrain	0.53727558	0.399713988	-1.4228714	2
81	Postcentral_L	0.52553897	0.332992653	-0.317387908	1
82	Occipital_Sup_R	0.522501286	0.375968499	-1.242502982	1
83	Precentral_R	0.521512829	0.337349962	-0.485939556	2
84	Midbrain	0.51916125	0.454768214	-1.393945971	1
85	SupraMarginal_R	0.518996924	0.319905905	-0.185804828	1
86	Parietal Inf_R	0.513079098	0.33479285	-0.604090662	2
87	Thalamus_R	0.509204671	0.376659107	-1.523720063	3
88	Lingual_BIL	0.505338366	0.319742756	-0.457133845	1
89	Paracentral_Lobule_BIL	0.503114286	0.350230544	-1.114815231	1
90	Postcentral_BIL	0.503097943	0.327131223	-0.650725402	2
91	Temporal_Mid_BIL	0.501047122	0.337169126	-0.893772636	2
92	Pons	0.500379445	0.403477024	-1.240335576	1
93	Occipital_Sup_R	0.499012248	0.312912064	-0.446989249	2
94	Postcentral_R	0.496588428	0.316175691	-0.561336984	2
95	Occipital_Mid_R	0.496150775	0.311384889	-0.473815674	2
96	Temporal_Sup_BIL	0.484510726	0.307651196	-0.632775203	1
97	Occipital_Sup_BIL	0.481284986	0.306194976	-0.668351912	1
98	Occipital_Sup_L	0.467186012	0.29239596	-0.674382642	1
99	Fusiform_BIL	0.457127764	0.258010197	-0.18527107	2
100	SupraMarginal_L	0.454337824	0.321235356	-0.342712047	1

***Supplementary table 2: C.elegans* network:** Full set of nodal results for PC_{norm} and PC, and z-score differences between PC_{norm} and PC. Note we used a z-score since PC_{norm} tends to yield higher values than PC -- see Fig. 2 and 3). The last column displays the module community affiliation for each node. See https://www.wormatlas.org/neurons/Individual%20Neurons/Neuronframeset.html for full naming, definition and function of specific *C.elegans* neurons.

	Node	PC_{norm}	PC	Z-score difference	Module
1	AVER	0.87188934	0.754524082	0.803904556	3
2	AVAR	0.850417827	0.751160024	0.648866855	3
3	AVAL	0.841479159	0.753069215	0.55598649	4
4	AVBR	0.832115473	0.744365741	0.550333698	4
5	AVBL	0.800101977	0.737799184	0.332454664	4
6	AVEL	0.799190253	0.697926538	0.666041661	3
7	AVDR	0.745524256	0.677048907	0.385304649	4
8	SAADR	0.726844052	0.7234	-0.171499293	5
9	AVDL	0.725415034	0.671363905	0.261803152	4
10	RMGR	0.723789379	0.70277551	-0.021064905	5
11	RIMR	0.722040586	0.685944637	0.108069255	5
12	PVCL	0.717077872	0.6878912	0.048911393	4
13	PVCR	0.711504866	0.665739645	0.190858447	4
14	RIML	0.709353064	0.688648889	-0.023716529	5
15	SMBDR	0.709172506	0.67614876	0.081764764	5
16	RIGL	0.698700791	0.68998	-0.126319365	2
17	SMBDL	0.691542967	0.6649375	0.026810875	5
18	RIGR	0.682752057	0.69403125	-0.297561068	2
19	RIAR	0.68186209	0.578097633	0.687453241	5
20	RIAL	0.678463093	0.587951389	0.573981956	5
21	AVJL	0.673746679	0.59897	0.439257217	4
22	AIBR	0.672858478	0.6596512	-0.087905654	1
23	AVJR	0.667032021	0.605767313	0.323566479	4
24	SAADL	0.653323653	0.706081633	-0.652706254	5
25	RIS	0.647143517	0.571755102	0.444494964	5
26	ADER	0.646803824	0.616611111	0.057525203	4
27	RIBL	0.643620868	0.55365625	0.569297749	2
28	RMFL	0.641424566	0.637591837	-0.168171398	5
29	DD2	0.641409023	0.603093333	0.127074877	3
30	DVA	0.64124183	0.653731405	-0.307924469	4
31	VB1	0.640809319	0.72224	-0.898204556	3
32	ADEL	0.631312445	0.60216	0.048618337	5
33	AVL	0.627692227	0.54575	0.500609304	4
34	AVKR	0.62530564	0.619263218	-0.149251809	5
35	ADAR	0.623203242	0.62355102	-0.203965293	5
36	SABD	0.621880552	0.570958678	0.235010189	3
37	SMBVL	0.617380281	0.65644898	-0.53549737	1
38	ADAL	0.612775816	0.621888889	-0.279014544	1
39	URXL	0.612358544	0.553033033	0.306962901	2
40	RIBR	0.612235515	0.499446192	0.764724973	5
41	AVKL	0.611602845	0.576077775	0.10318133	5
42	BDUR	0.610350967	0.542333333	0.381385657	4
43	BDUL	0.605318518	0.583833834	-0.01703374	4
44	DVC	0.603067698	0.5429	0.314173784	2
45	RMHL	0.603062953	0.535341365	0.378850876	5
46	RIPL	0.602471823	0.438602041	1.202080562	5
47	DA8	0.601391266	0.578760331	-0.007219429	6
48	AQR	0.601340044	0.607888889	-0.257059395	4
49	RIR	0.599914812	0.612975207	-0.312811881	2
50	FLPL	0.598544558	0.548136273	0.230612799	4
51	BAGR	0.594557278	0.597375	-0.225113178	2
52	SABVL	0.593533733	0.549111111	0.179362994	3
53	AUAR	0.592592963	0.62944	-0.516475302	5
54	OLLR	0.590665538	0.46525	0.872831786	5
55	RMGL	0.587483553	0.468	0.822041588	5
56	RMHR	0.585681016	0.45622582	0.907419706	5
57	AUAL	0.584712572	0.585877551	-0.210962239	2
58	SIBVL	0.580950739	0.465611111	0.786560917	5
59	RMEV	0.578849947	0.425003516	1.116259707	5
60	AIZL	0.574583317	0.579289256	-0.241280263	1
61	SMBVR	0.574003944	0.607020408	-0.483677579	1
62	ALML	0.573906115	0.539070352	0.097279431	5
63	ADLL	0.573456303	0.549777778	0.001750134	4
64	PDEL	0.571604532	0.603985749	-0.478238541	4
65	OLQVL	0.571197681	0.4575	0.772502418	5
66	DD1	0.568701325	0.484648711	0.51867863	3

67	AS5	0.568014997	0.449975309	0.809679088	3
68	DB5	0.56761642	0.538055556	0.052115255	6
69	VD1	0.56744537	0.483398955	0.518625556	3
70	RIH	0.567310708	0.38504142	1.359618818	5
71	DD4	0.564536145	0.568319444	-0.233380539	6
72	URXR	0.562172023	0.457936281	0.691488427	5
73	AS1	0.561330095	0.461497155	0.653791231	3
74	DA5	0.559985812	0.43142	0.899804722	3
75	RMDR	0.559553639	0.339638917	1.681942488	5
76	SABVR	0.558746515	0.50474059	0.261416113	4
77	DD3	0.558440096	0.48624	0.417196279	3
78	VA12	0.558421336	0.530868059	0.034926115	6
79	HSNR	0.557181027	0.468773333	0.555967219	3
80	SMDDL	0.556831482	0.344183176	1.619726764	5
81	DVB	0.553558696	0.49321875	0.315648585	2
82	RIPR	0.552366404	0.340485207	1.613158708	5
83	VD4	0.551797376	0.447641563	0.690804075	3
84	SAAVR	0.551321787	0.491310685	0.312832994	5
85	PVQL	0.547616604	0.445027778	0.677387385	1
86	HSNL	0.546800579	0.460066116	0.541640865	3
87	ASGR	0.544494291	0.582125	-0.523185169	1
88	ASHR	0.542021813	0.550428217	-0.272963991	1
89	PVPR	0.540580246	0.493234806	0.204388427	4
90	VA6	0.540023105	0.42489403	0.784758149	3
91	DB3	0.539552472	0.49930203	0.143640402	3
92	PVPL	0.539434165	0.489567839	0.225972486	4
93	DA6	0.53613316	0.522400535	-0.083407585	6
94	ASKL	0.535892447	0.47230263	0.343474281	1
95	RMED	0.535103318	0.42352	0.754399052	5
96	RIFL	0.531020525	0.52492485	-0.148795844	4
97	AWBL	0.530759057	0.555173293	-0.410024511	1
98	ADFR	0.530274365	0.499625	0.061435097	1
99	DA2	0.530214902	0.426519916	0.686858422	3
100	PQR	0.530142425	0.479362912	0.233791275	4
101	AIZR	0.529796473	0.500115702	0.053141906	1
102	PVWL	0.527370939	0.488526077	0.131605699	4
103	SIBVR	0.525853725	0.375	1.090635879	5
104	PVR	0.525498708	0.47437037	0.236777947	4
105	AVHR	0.523937006	0.403024793	0.834273903	4
106	VD3	0.518441763	0.393358903	0.869983373	3
107	PHBL	0.517129113	0.496849943	-0.027355456	4
108	VA11	0.516995245	0.429906932	0.544670561	6
109	DA9	0.515226962	0.491970339	-0.001862233	6
110	URADL	0.514979632	0.445364619	0.39506256	5
111	URADR	0.514342	0.444444444	0.397481713	5
112	VA10	0.514313513	0.459166667	0.271184787	6
113	PDER	0.513137744	0.499498998	-0.084211385	4
114	VD6	0.512309061	0.446779853	0.360079527	3
115	PVNR	0.51048801	0.39753125	0.766158582	4
116	RMFR	0.506886964	0.427402863	0.479562598	5
117	RIFR	0.50632863	0.403807615	0.676806775	4
118	VD12	0.502386789	0.480147796	-0.010575271	6
119	AS10	0.50131257	0.38604345	0.785957227	6
120	CEPDR	0.496916502	0.281685776	1.641837686	5
121	SMDDR	0.49607419	0.330631118	1.215551215	5
122	AIAL	0.493456509	0.411572667	0.500109404	1
123	RICL	0.493161228	0.257777778	1.814387165	5
124	SMDVR	0.491356161	0.249104283	1.873195269	5
125	PVQR	0.491174759	0.348625	1.019536559	4
126	LUAL	0.489301321	0.503755669	-0.324747033	4
127	VD5	0.487420582	0.360851927	0.882704882	3
128	IL1DR	0.483364612	0.32024	1.19570035	5
129	IL1VR	0.48249764	0.278	1.549940016	5
130	IL1DL	0.481883626	0.32088	1.17754027	5
131	VA5	0.479996679	0.33698092	1.023526496	3
132	PVWR	0.479617162	0.446578631	0.081891359	4
133	IL1VL	0.479509647	0.277777778	1.526259236	5
134	AS2	0.479434762	0.461061224	-0.043671663	3
135	RMDL	0.477765481	0.243496311	1.804846595	5
136	VC2	0.477676492	0.345485436	0.930844401	3
137	DA1	0.4772101	0.327350622	1.082123042	3
138	RICR	0.474975069	0.177384615	2.347009249	5
139	DB4	0.474442485	0.429154775	0.186769958	3
140	ALNL	0.473633357	0.5	-0.426741205	5
141	VC5	0.471821577	0.448022312	0.002783909	2

142	SIAVR	0.471641456	0.341552941	0.912842246	5
143	VA4	0.47062233	0.494707151	-0.407204034	3
144	RID	0.470080085	0.4485	-0.016216905	4
145	AVM	0.463153439	0.515655024	-0.650510981	4
146	AS3	0.463112168	0.425167858	0.123895093	3
147	AS6	0.461302801	0.474675768	-0.315488151	3
148	AS4	0.460658081	0.425164332	0.102913157	3
149	DD6	0.456221029	0.402163265	0.261859961	6
150	AWBR	0.453413318	0.469518717	-0.338883486	1
151	PVNL	0.451690583	0.297323961	1.120713633	4
152	DA3	0.45163069	0.310834958	1.004518428	3
153	AIAR	0.450658601	0.368107873	0.505819343	1
154	PVT	0.449925255	0.445553603	-0.16355709	4
155	ASHL	0.449880197	0.47341954	-0.402533602	4
156	LUAR	0.447620159	0.48102981	-0.487044084	4
157	ALNR	0.445121312	0.5	-0.670863954	5
158	AS11	0.445086132	0.443406769	-0.186608722	6
159	AVG	0.44275169	0.268336155	1.292374265	4
160	SAAVL	0.44271288	0.395946924	0.199426839	5
161	FLPR	0.439357681	0.463477366	-0.407502541	4
162	DA7	0.438185466	0.478541667	-0.54652108	6
163	PHBR	0.436598306	0.444444444	-0.268166936	4
164	VD7	0.434131652	0.377681452	0.28234423	6
165	RIVR	0.430167614	0.338916968	0.580308845	5
166	VD13	0.428631317	0.483376793	-0.66972338	6
167	DB7	0.427679625	0.461374002	-0.489481939	6
168	BAGL	0.424696177	0.5	-0.845745838	2
169	RMDDL	0.424310334	0.111774281	2.474974843	5
170	RMDVL	0.422332494	0.133219373	2.274425545	5
171	AVFL	0.422312095	0.29202542	0.914538904	4
172	AVFR	0.42230374	0.287269272	0.955189951	4
173	VD11	0.420540594	0.339258517	0.494957038	6
174	RMDVR	0.419663322	0.135252214	2.234166475	5
175	RMDDR	0.418713682	0.130752688	2.264560922	5
176	VA2	0.417048118	0.409914894	-0.139912257	3
177	OLLL	0.414696331	0.381831984	0.080399982	5
178	VA3	0.414514614	0.400199468	-0.078419985	3
179	ADLR	0.414458706	0.444444444	-0.457728232	1
180	CEPDL	0.414303209	0.281959184	0.93215413	5
181	DA4	0.409441611	0.417331046	-0.26853765	3
182	RMER	0.408041979	0.152770307	1.984671951	5
183	AVHL	0.407937705	0.282648346	0.871751431	4
184	VA7	0.402236119	0.446115288	-0.576685065	6
185	RMEL	0.394885938	0.197530864	1.488784708	5
186	VD2	0.394026186	0.191012366	1.537235413	3
187	SIADR	0.387009331	0.447222222	-0.716535893	5
188	IL1L	0.385153956	0.296052632	0.561906148	5
189	CEPVL	0.38130192	0.392487047	-0.296755665	5
190	CEPVR	0.378950796	0.337731959	0.151931896	5
191	IL1R	0.376533816	0.277777778	0.644570699	5
192	URAVR	0.375411122	0	-0.20098758	5
193	IL2VR	0.374876677	0	-0.20098758	5
194	IL2R	0.374634623	0.444444444	-0.798705689	5
195	SIADL	0.37440658	0.322147651	0.246458176	5
196	ASER	0.369885751	0.277042426	0.593945505	1
197	VC3	0.369613715	0.469886364	-1.059531217	3
198	OLQVR	0.366518775	0.32	0.197310452	5
199	RIVL	0.361920141	0.37962963	-0.352617847	5
200	AWAR	0.357515432	0.375775434	-0.357331395	1
201	SIAVL	0.357490922	0.338823529	-0.041155645	5
202	AIYR	0.354852994	0.248457879	0.709977171	1
203	ASJL	0.353740557	0.5	-1.453274378	1
204	OLQDL	0.353269029	0.32	0.083864907	5
205	OLQDR	0.35298403	0	-0.20098758	5
206	VA9	0.351475232	0.444444444	-0.996998529	6
207	DB2	0.347664165	0.500825991	-1.512373214	3
208	ASGL	0.337357905	0.444818556	-1.121075554	1
209	VB11	0.336909189	0.445919371	-1.134342777	6
210	PVM	0.330227981	0.43772482	-1.121385403	4
211	VA1	0.323157532	0.492021277	-1.646814484	3
212	DD5	0.32032843	0.279584639	0.147864504	6
213	VD10	0.310794679	0.286743269	0.004942807	6
214	AWCR	0.304949476	0.245633388	0.306882218	1
215	VD9	0.297524929	0.259381607	0.125599051	6
216	ASKR	0.29665875	0.444444444	-1.466342284	1

217	ASEL	0.282261127	0.361111111	-0.876108391	1
218	ADFL	0.279710673	0.444444444	-1.611453277	1
219	VD8	0.266062576	0.282418672	-0.341029974	6
220	SIBDR	0.264173524	0.5	-1.220155553	5
221	URYVR	0.255295101	0.5	-1.296173428	5
222	URBL	0.251369812	0.5	-1.329782113	5
223	AWCL	0.249379649	0	-0.20098758	1
224	URYDL	0.248174522	0.5	-2.357140475	5
225	URAVL	0.241037027	0	-0.20098758	5
226	URYDR	0.237559785	0	-0.20098758	5
227	URBR	0.234631582	0	-0.20098758	5
228	VC4	0.219372325	0.5	-1.603747531	2
229	AINR	0.207559545	0.5	-1.704889644	1
230	VB5	0.201323271	0.5	-1.758285199	4
231	VB7	0.20059122	0.5	-1.764553086	4
232	AFDR	0.20001531	0.444444444	-1.293812303	1
233	VB3	0.198212055	0.5	-1.784923716	4
234	VB6	0.197846029	0.5	-1.788057659	4
235	AFDL	0.197539343	0.444444444	-1.315011758	1
236	VB8	0.197480004	0.5	-1.791191603	6
237	VB4	0.195466864	0.5	-1.80842829	4
238	ASJR	0.184880836	0.5	-1.899066839	3
239	PHAL	0.183571038	0.5	-1.910281443	4
240	PHAR	0.177172771	0.5	-1.965063997	4
241	VB2	0.158864324	0.5	-1.121822606	3
242	AIML	0.140093475	0.5	-1.282540341	1
243	AIMR	0	0	-0.20098758	1
244	AINL	0	0	-0.20098758	1
245	ALA	0	0	-0.20098758	3
246	ALMR	0	0	-0.20098758	5
247	AS7	0	0	-0.20098758	6
248	AS8	0	0	-0.20098758	6
249	AS9	0	0	-0.20098758	6
250	ASIL	0	0	-0.20098758	1
251	ASIR	0	0	-0.20098758	1
252	AWAL	0	0	-0.20098758	1
253	DB1	0	0	-0.20098758	3
254	DB6	0	0	-0.20098758	6
255	IL2DL	0	0	-0.20098758	5
256	IL2DR	0	0	-0.20098758	5
257	IL2L	0	0	-0.20098758	5
258	IL2VL	0	0	-0.20098758	5
259	PDA	0	0	-0.20098758	6
260	PDB	0	0	-0.20098758	6
261	PHCL	0	0	-0.20098758	4
262	PHCR	0	0	-0.20098758	4
263	PLML	0	0	-0.20098758	3
264	PLMR	0	0	-0.20098758	4
265	PLNL	0	0	-0.20098758	1
266	PLNR	0	0	-0.20098758	1
267	PVDL	0	0	-0.20098758	4
268	PVDR	0	0	-0.20098758	4
269	SDQL	0	0	-0.20098758	5
270	SDQR	0	0	-0.20098758	4
271	SIBDL	0	0	-0.20098758	5
272	URYVL	0	0	-0.20098758	5
273	VA8	0	0	-0.20098758	6
274	VB10	0	0	-0.20098758	6
275	VB9	0	0	-0.20098758	6
276	VC1	0	0	-0.20098758	3
277	VC6	0	0	-0.20098758	6

Supplementary table 3: **airport network:** Full set of nodal results for PC_{norm} and PC, and z-score differences between PC_{norm} and PC. Note we used a z-score since PC_{norm} tends to yield higher values than PC -- see Fig. 2 and 3). The last column displays the module community affiliation for each node. See https://en.wikipedia.org/wiki/List_of_airports_by_IATA_code:_A for full naming of specific airports.

	Node	PC_{norm}	PC	Z-score difference	Module
1	JFK	0.853633188	0.60689782	-0.400287705	3
2	PUJ	0.830163416	0.57885355	-0.371498864	3
3	YYZ	0.813662563	0.546042274	-0.268851938	3
4	YUL	0.806824245	0.49104939	0.034200768	3
5	FRA	0.781019975	0.620267069	-0.941403954	4
6	EWR	0.763787078	0.489687189	-0.228073664	3
7	YVR	0.759960001	0.461996037	-0.077889097	3
8	LHR	0.740896613	0.565572015	-0.849699454	4
9	HAV	0.740439574	0.66026847	-1.448532061	1
10	LAX	0.737213446	0.48138189	-0.343042365	3
11	ANU	0.73664054	0.489695	-0.398965018	3
12	AMS	0.733367541	0.575281867	-0.958189734	4
13	ORD	0.73087708	0.457365182	-0.231774091	3
14	ATL	0.730516484	0.470233554	-0.31502839	3
15	AUA	0.728819252	0.497691327	-0.498510531	3
16	ANC	0.724650218	0.425703889	-0.071706744	3
17	CUN	0.723621187	0.406816339	0.040682859	3
18	CDG	0.723058694	0.5743955	-1.017488536	4
19	CCS	0.721008886	0.657781664	-1.555165546	1
20	MBJ	0.706552477	0.347093333	0.309120538	3
21	SFO	0.706492498	0.413808219	-0.111115914	3
22	HNL	0.703866349	0.4584	-0.408274061	3
23	SDQ	0.703012365	0.574502058	-1.144317362	1
24	BOS	0.701639925	0.381394276	0.062336961	3
25	BGI	0.700275671	0.450151229	-0.378959132	3
26	IAD	0.699237515	0.409190336	-0.127712061	3
27	YYT	0.697515204	0.32	0.422753337	3
28	SJO	0.690855782	0.557470679	-1.113638648	1
29	MIA	0.69019384	0.475650722	-0.602884249	3
30	YHZ	0.686946028	0.301932183	0.469944779	3
31	MEX	0.683700028	0.428855443	-0.349253702	3
32	LUX	0.68362019	0.540977366	-1.055376731	4
33	SNN	0.680027083	0.408163265	-0.242146007	4
34	SXM	0.67787131	0.403077289	-0.223705263	3
35	YYC	0.677839803	0.29579	0.451291074	3
36	FCO	0.674138605	0.491871802	-0.806009846	4
37	LGW	0.673991543	0.435184807	-0.450185223	4
38	KIN	0.673323422	0.38738	-0.153538367	3
39	MAN	0.671852108	0.45499811	-0.588341113	4
40	PHL	0.671366346	0.328922902	0.202035072	3
41	DKR	0.670016374	0.57897503	-1.380122016	4
42	PPT	0.669404567	0.666716049	-1.936155764	2
43	GUA	0.668459495	0.397475995	-0.247686137	3
44	NRT	0.665688326	0.579870762	-1.412997007	2
45	MXP	0.663255996	0.501962111	-0.937999395	4
46	LIM	0.659860919	0.615192508	-1.671962319	1
47	BOG	0.658190777	0.62934375	-1.771531557	1
48	SJU	0.655545474	0.39881893	-0.337409905	3
49	AMM	0.654699496	0.543360947	-1.252384951	2
50	PTP	0.653054231	0.615306122	-1.715514087	1
51	MUC	0.652570314	0.443796433	-0.639191975	4
52	SAL	0.650267437	0.379867036	-0.251355775	3
53	LOS	0.650004419	0.53888	-1.253732545	2
54	TPE	0.647088557	0.569208045	-1.462947514	2
55	MAD	0.645046125	0.477911428	-0.901241222	4
56	ZRH	0.644952819	0.448945024	-0.719533207	4
57	EZE	0.643612769	0.680514178	-2.185308348	1
58	ATH	0.637529017	0.434879027	-0.677731661	4
59	IAH	0.637485531	0.322421904	0.029724769	3
60	SCL	0.636552754	0.666834977	-2.143651612	1
61	TLV	0.635947426	0.435840666	-0.693737053	4
62	GDL	0.634109853	0.288597463	0.22134898	3
63	SVO	0.633321295	0.457599683	-0.847200904	4
64	ACC	0.632855216	0.495907407	-1.09121736	2
65	YOW	0.630939249	0.20244898	0.74355639	3
66	DME	0.630731409	0.505613169	-1.165664772	4

67	JNB	0.629935391	0.565163422	-1.545443941	2
68	DXB	0.627265397	0.53632822	-1.380777578	2
69	YEG	0.627125566	0.205459566	0.700608993	3
70	SEA	0.626845434	0.245762446	0.445206586	3
71	GLA	0.624229958	0.351869684	-0.239021643	4
72	CAI	0.619015163	0.506659408	-1.245983338	2
73	DTW	0.616467299	0.246489708	0.375316628	3
74	MCO	0.61546011	0.22948045	0.476022972	3
75	CLO	0.614503311	0.548260355	-1.536186529	1
76	POS	0.614384875	0.288262976	0.09931816	3
77	GYE	0.613677975	0.605783934	-1.90339567	1
78	GRU	0.610446384	0.654003693	-2.22719615	1
79	IST	0.60923217	0.44302663	-0.907088719	4
80	BRU	0.608008059	0.380012432	-0.518223128	4
81	DFW	0.607207222	0.25516954	0.262414804	3
82	ICN	0.607152358	0.528077916	-1.455433714	2
83	DEL	0.604527279	0.530731864	-1.488656392	2
84	MGA	0.602100534	0.366826531	-0.472417886	3
85	BCN	0.601929679	0.399775047	-0.680849114	4
86	ISB	0.599900709	0.544880658	-1.60681601	2
87	PEK	0.59788337	0.524086723	-1.488648642	2
88	THR	0.595746981	0.509918835	-1.412930407	4
89	PTY	0.594401858	0.53565023	-1.583331954	1
90	NAS	0.593763144	0.161346483	0.768266483	3
91	FAI	0.590792882	0.132653061	0.930150882	3
92	LAS	0.589382777	0.176970223	0.642373972	3
93	BEY	0.586119668	0.465341784	-1.192980074	2
94	PDX	0.58584412	0.159745775	0.728503211	3
95	DAM	0.585626898	0.442614379	-1.053050115	4
96	CVG	0.584808868	0.160763393	0.715583818	3
97	UIO	0.581493537	0.604785124	-2.099657214	1
98	CPT	0.580276743	0.521137778	-1.580894307	2
99	SAN	0.579629063	0.135401916	0.842593801	3
100	DEN	0.579431318	0.158975026	0.692995895	3
101	CLT	0.579246311	0.153949246	0.723460495	3
102	JED	0.579026866	0.503803571	-1.479670268	2
103	DUS	0.57684007	0.303305136	-0.231629109	4
104	VIE	0.573485126	0.331752041	-0.431768735	4
105	PVR	0.572438829	0.099774376	1.021559228	3
106	KOA	0.572192104	0.124444444	0.864749556	3
107	KBP	0.5707903	0.344165183	-0.526848211	4
108	LIS	0.570129046	0.357509861	-0.6149922	4
109	FLL	0.57004278	0.154775034	0.660342646	3
110	YWG	0.569946024	0.097279429	1.021572708	3
111	CMN	0.569071254	0.355283731	-0.607639459	4
112	TIJ	0.568181222	0.129695502	0.80646109	3
113	LHE	0.567905173	0.510005102	-1.588691082	2
114	VER	0.566727442	0.177520661	0.49633232	3
115	CPH	0.565018254	0.316813795	-0.391042222	4
116	ARN	0.564741213	0.320699085	-0.417237164	4
117	MRU	0.563798069	0.4847392	-1.455531724	2
118	TPA	0.563092565	0.115225318	0.865502161	3
119	DUB	0.562989348	0.274289632	-0.136192095	4
120	DOH	0.56267188	0.479936728	-1.432395652	2
121	MLE	0.562406524	0.492561983	-1.513520561	2
122	HEL	0.561578683	0.331910431	-0.507696746	4
123	BWI	0.561403831	0.127818559	0.775620938	3
124	AUH	0.561318452	0.483810403	-1.465291553	2
125	HKG	0.561306478	0.470259695	-1.380087792	2
126	LEJ	0.557918384	0.333596735	-0.541344698	4
127	ADD	0.557871375	0.472101563	-1.413297525	2
128	MTY	0.557768712	0.073525952	1.094425372	3
129	NBO	0.557692106	0.456320499	-1.315110241	2
130	BOM	0.556004751	0.476612903	-1.453436176	2
131	BKK	0.555685644	0.470869168	-1.419297185	2
132	PVG	0.554187617	0.471761342	-1.434339516	2
133	ALG	0.553341239	0.331348026	-0.555998318	4
134	KEF	0.552298546	0.244897959	-0.018501291	4
135	RSW	0.550612121	0.063657943	1.111489224	3
136	PHX	0.550595074	0.089644968	0.947836946	3
137	KHI	0.54966308	0.483908173	-1.539257985	2
138	SIN	0.549356082	0.457539571	-1.375243635	2
139	FDF	0.548542024	0.571277778	-2.096159168	1
140	SLC	0.547871642	0.076394547	1.01408679	3
141	STT	0.545805263	0.069695291	1.043243031	3

142	MSP	0.545571496	0.090920911	0.908191959	3
143	KWI	0.544577436	0.467057556	-1.465217094	2
144	SYR	0.544498888	0.06795728	1.045959456	3
145	LGA	0.544234184	0.091413277	0.89667719	3
146	RDU	0.543636149	0.063954086	1.065723382	3
147	MSY	0.541812766	0.053409578	1.12060833	3
148	TIP	0.540198455	0.332051111	-0.643134983	4
149	DCA	0.539801054	0.072266446	0.989275406	3
150	ORY	0.539631824	0.282372191	-0.334055002	4
151	ABJ	0.538950592	0.362903047	-0.845149704	2
152	MEL	0.535850583	0.460123269	-1.476498307	2
153	SAT	0.534638146	0.047551856	1.112320646	3
154	BDL	0.534598133	0.04648038	1.11881199	3
155	MID	0.534593696	0.045207101	1.126797237	3
156	OPO	0.533875592	0.272040763	-0.30526177	4
157	MZT	0.532097059	0.017822314	1.28342665	3
158	RUH	0.532061412	0.453827977	-1.460726456	2
159	DLA	0.53191804	0.391916667	-1.07200026	2
160	BJX	0.53166471	0.018643599	1.275537116	3
161	BIL	0.531182868	0.0129792	1.30815267	3
162	RIC	0.531143659	0.033030965	1.181713485	3
163	ACA	0.530967142	0.017826531	1.276289165	3
164	TLH	0.530003753	0.009648789	1.321691493	3
165	PNS	0.529612231	0.010232422	1.315554518	3
166	BNA	0.529037237	0.045286547	1.091328608	3
167	XNA	0.52895103	0.011646457	1.302494362	3
168	MFE	0.528869898	0.006654064	1.333402565	3
169	SAV	0.528615395	0.010087302	1.310194385	3
170	HSV	0.528504571	0.009443787	1.313546783	3
171	FAT	0.528393986	0.001814059	1.360867252	3
172	EMA	0.528293399	0.228688264	-0.067560663	4
173	TYS	0.528279964	0.008994646	1.314959854	3
174	SMF	0.528093737	0.025246079	1.211512189	3
175	MEM	0.528007976	0.029318534	1.185343148	3
176	LEX	0.527979087	0.001141869	1.362486477	3
177	BZN	0.527878508	0	1.369039658	3
178	CLE	0.527785989	0.036158416	1.140900456	3
179	CUU	0.527747744	0.022663265	1.225589243	3
180	SJD	0.527739206	0.007603175	1.320313674	3
181	FSD	0.527705384	0	1.367950127	3
182	CID	0.527691089	0	1.367860165	3
183	BTR	0.527672009	0	1.367740088	3
184	FNT	0.527599129	0	1.367281433	3
185	CRP	0.52758151	0	1.367170547	3
186	PVD	0.527506647	0.030069197	1.177463945	3
187	IND	0.527360659	0.026371165	1.199818138	3
188	MLI	0.527338989	0	1.365644283	3
189	ICT	0.527274871	0.004099723	1.339439846	3
190	OGG	0.527271604	0	1.365220209	3
191	MYR	0.527261335	0	1.365155581	3
192	FAR	0.527208016	0	1.36482003	3
193	VPS	0.527176809	0.001426612	1.35564549	3
194	LGB	0.527108723	0	1.364195143	3
195	SDF	0.527075829	0.028288281	1.185960564	3
196	OMA	0.526921951	0.026824229	1.194205922	3
197	AKL	0.5268456	0.495513672	-1.755893248	2
198	HPN	0.526783256	0	1.362146869	3
199	GRB	0.526736534	0	1.361852834	3
200	MDT	0.526701203	0	1.361630485	3
201	PSP	0.526654239	0	1.361334924	3
202	SBA	0.52661251	0	1.361072309	3
203	GSP	0.526563387	0.007871435	1.311225603	3
204	COS	0.526532179	0.000975	1.35443076	3
205	BTV	0.526394507	0	1.359700347	3
206	DSM	0.526161817	0	1.358235948	3
207	GSO	0.526067814	0.006894965	1.314252045	3
208	MSN	0.525952277	0	1.356917245	3
209	SBN	0.525939447	0	1.356836501	3
210	SRQ	0.525930743	0	1.356781726	3
211	CAE	0.525887109	0	1.35650712	3
212	CHS	0.525833056	0.006143347	1.317504818	3
213	BUF	0.525792577	0.025655845	1.194451426	3
214	YYJ	0.525718444	0	1.355445652	3
215	PWM	0.525377804	0	1.353301893	3
216	ISP	0.525209878	0	1.352245082	3

217	GRR	0.525007404	0	1.350970845	3
218	DAY	0.52481538	0.003742604	1.32620892	3
219	EUG	0.524808744	0	1.349720608	3
220	HMO	0.524743471	0.020153061	1.222479904	3
221	MAF	0.524589243	0	1.348339216	3
222	LBB	0.524489163	0	1.347709378	3
223	AMA	0.524440283	0	1.347401765	3
224	HRL	0.524245586	0	1.346176467	3
225	ROC	0.524087751	0	1.345183158	3
226	BOI	0.523690802	0.001206525	1.335091965	3
227	AUS	0.523592064	0.024067924	1.190596185	3
228	TUS	0.523471729	0.008158861	1.289959899	3
229	STL	0.523057915	0.029248438	1.154631908	3
230	BUR	0.52302258	0.000677765	1.334214289	3
231	GEG	0.52290546	0	1.337742609	3
232	CJS	0.522874675	0.020958678	1.205648933	3
233	LIH	0.522819092	0	1.337199066	3
234	SNA	0.522811663	0.010168006	1.273161672	3
235	PBI	0.522808102	0	1.337129905	3
236	ALB	0.522699816	0.00519873	1.303731081	3
237	TUN	0.522399999	0.275165406	-0.39714592	4
238	JAN	0.522035545	0.000644988	1.328208821	3
239	RNO	0.521971175	0.000634755	1.327868116	3
240	LIT	0.521917055	0.000624843	1.327589905	3
241	DAL	0.520640193	0.000596878	1.319730181	3
242	MKE	0.520589177	0.002687183	1.306254129	3
243	ONT	0.52038151	0.003197598	1.301735002	3
244	ELP	0.520142886	0	1.320356812	3
245	CUL	0.520108617	0.0249375	1.163201157	3
246	YLW	0.520091472	0	1.32003325	3
247	MHT	0.519917078	0.004332602	1.291669216	3
248	MDW	0.519913152	0.00989282	1.256652212	3
249	BHM	0.519878602	0.004224323	1.292108517	3
250	HOU	0.519861263	0	1.318584467	3
251	JAX	0.519855869	0.004845336	1.288057203	3
252	VSA	0.519778702	0.0219	1.180240891	3
253	PIT	0.519657577	0.017625573	1.206379009	3
254	ORF	0.519308417	0.004784024	1.284997765	3
255	TUL	0.519219663	0	1.314546661	3
256	OKC	0.519046363	0.002024984	1.300712131	3
257	SJC	0.518082596	0.000963268	1.301328558	3
258	ABQ	0.517263843	0.000470522	1.299276886	3
259	OAK	0.517030269	0.000919031	1.294984308	3
260	MCI	0.515543005	0.007817762	1.242208447	3
261	STN	0.515413105	0.21736263	-0.077344655	4
262	CMH	0.515314534	0.003448613	1.268267117	3
263	PRG	0.512646511	0.197140788	0.032507036	4
264	KRT	0.511562432	0.433185596	-1.459823983	2
265	JNU	0.511365239	0	1.265116155	3
266	EDI	0.511097491	0.176855056	0.150423396	4
267	SAH	0.510726485	0.434321361	-1.472232628	2
268	OTP	0.509987733	0.208818182	-0.057715272	4
269	MDE	0.507787248	0.476946746	-1.758985956	1
270	GVA	0.507028551	0.209686166	-0.081800901	4
271	LCA	0.505756365	0.23154479	-0.227370792	4
272	SYD	0.505702161	0.432394464	-1.491725761	2
273	TXL	0.505156259	0.194461813	0.002228064	4
274	BFS	0.504929006	0.165289256	0.184390494	4
275	DAR	0.504748372	0.427555556	-1.467275414	2
276	SHJ	0.50397869	0.415764938	-1.397917001	2
277	VCE	0.503131107	0.168628166	0.152062846	4
278	ITO	0.503036956	0	1.212703502	3
279	CGN	0.495071791	0.180208148	0.028466207	4
280	BUD	0.494885017	0.178245825	0.03964033	4
281	MRS	0.494304634	0.16861678	0.096586568	4
282	KTN	0.493564418	0	1.153089668	3
283	GIG	0.492964201	0.530649796	-2.190243493	1
284	NTE	0.490282283	0.196270408	-0.102760907	4
285	LED	0.489744926	0.193761815	-0.09035526	4
286	GOT	0.487683558	0.171088156	0.039364748	4
287	KIX	0.483279843	0.378138184	-1.291384042	2
288	NAN	0.482907438	0.397338843	-1.414563853	2
289	NGO	0.481496695	0.379060764	-1.30841209	2
290	MAA	0.481193092	0.3871025	-1.360932085	2
291	BHX	0.480177269	0.141078343	0.180986909	4

292	KRK	0.479678092	0.121928166	0.29836386	4
293	VVI	0.477749255	0.51752	-2.203366035	1
294	CWL	0.47707454	0.120707596	0.289660292	4
295	BAH	0.475560987	0.375428701	-1.322909693	2
296	LYS	0.47355634	0.120027778	0.271797413	4
297	BSL	0.472879701	0.148433153	0.088774618	4
298	BLQ	0.472676645	0.123766541	0.242731923	4
299	WAW	0.471799355	0.103584162	0.364225268	4
300	DMM	0.471430306	0.369556787	-1.311951538	2
301	MNL	0.46865091	0.35544186	-1.240613273	2
302	SUF	0.468023408	0.104938272	0.331940118	4
303	OSL	0.46787529	0.118248521	0.247242135	4
304	PSA	0.467446935	0.097304405	0.376354641	4
305	SGN	0.465465356	0.367220703	-1.334789197	2
306	HAM	0.464652734	0.096578433	0.36333859	4
307	NCE	0.461019932	0.092300219	0.367400383	4
308	KUL	0.459827138	0.34894727	-1.255271582	2
309	BOD	0.457223924	0.087621224	0.372957308	4
310	NCL	0.456460982	0.087693889	0.367698565	4
311	AMD	0.453698347	0.361259259	-1.371325555	2
312	HKT	0.452542819	0.3791875	-1.491426063	2
313	LTN	0.451028649	0.074504421	0.416516793	4
314	BEG	0.450264714	0.091402344	0.305364843	4
315	AGP	0.449079552	0.071611601	0.422455956	4
316	CMB	0.44879611	0.338685172	-1.260110714	2
317	PMO	0.4471646	0.060546875	0.480038532	4
318	TFN	0.445529042	0.110615917	0.154644269	4
319	TLS	0.445001925	0.055510204	0.498125546	4
320	STR	0.444255804	0.049351066	0.532191464	4
321	LPL	0.443866272	0.052593134	0.509336595	4
322	MVD	0.443667547	0.492663265	-2.261421869	1
323	SOF	0.443531965	0.069038399	0.403737159	4
324	SCQ	0.443447883	0.099623269	0.21072724	4
325	RIX	0.439486782	0.042533081	0.545086268	4
326	SXF	0.438010101	0.048895508	0.495752154	4
327	FAO	0.437588137	0.038446751	0.558854097	4
328	NAP	0.436177674	0.035702479	0.5672482	4
329	BRS	0.435270468	0.033888228	0.572956534	4
330	FNC	0.434069604	0.058710744	0.409182749	4
331	MLA	0.430182317	0.032295482	0.550958792	4
332	BLR	0.429720651	0.310866941	-1.205089546	2
333	ALC	0.428673673	0.030295858	0.554048691	4
334	LPA	0.422115553	0.009532562	0.643446594	4
335	PMI	0.419817173	0	0.688973707	4
336	IBZ	0.41912439	0	0.684613791	4
337	ACE	0.418771855	0	0.68239517	4
338	HAJ	0.418744975	0	0.682226006	4
339	TFS	0.418601211	0	0.681321252	4
340	FUE	0.418547057	0	0.680980443	4
341	LBA	0.418318465	0	0.679541836	4
342	VLC	0.418225729	0	0.678958219	4
343	HER	0.418214733	0	0.678889019	4
344	MAH	0.418143589	0	0.678441283	4
345	BGY	0.418051982	0	0.677864774	4
346	SVQ	0.41780421	0	0.676305459	4
347	NUE	0.417710432	0	0.675715283	4
348	CTA	0.417679609	0	0.675521304	4
349	ORK	0.417452397	0	0.674091387	4
350	GRO	0.417339674	0	0.673381982	4
351	SKG	0.417229108	0	0.672686153	4
352	LCY	0.417045738	0	0.671532146	4
353	BRE	0.417019633	0	0.671367854	4
354	SOU	0.416581533	0	0.66861075	4
355	VNO	0.416562284	0	0.668489609	4
356	ABZ	0.416484565	0	0.668000498	4
357	BLL	0.416452811	0	0.667800658	4
358	TRN	0.416437843	0	0.667706461	4
359	RAK	0.416420079	0	0.667594666	4
360	LIN	0.416373657	0	0.667302512	4
361	AYT	0.416344988	0	0.667122092	4
362	BIO	0.416165736	0	0.665994001	4
363	SZG	0.415899705	0	0.664319775	4
364	SVG	0.415881738	0	0.664206704	4
365	BGO	0.415852701	0	0.664023966	4
366	JER	0.415764901	0	0.663471411	4

367	VRN	0.415680446	0	0.662939909	4
368	LJU	0.415645762	0	0.662721631	4
369	TLL	0.415504428	0	0.661832167	4
370	ESB	0.415373354	0	0.661007276	4
371	ZAG	0.415353412	0	0.660881773	4
372	FLR	0.415279616	0	0.660417351	4
373	XRY	0.415245059	0	0.660199874	4
374	ADB	0.414846401	0	0.657690987	4
375	DRS	0.414806941	0	0.657442649	4
376	SXB	0.414523077	0	0.655656197	4
377	BRI	0.413861292	0	0.651491366	4
378	GRZ	0.413386331	0	0.648502275	4
379	MCT	0.41313852	0.275624567	-1.087654424	2
380	DAC	0.41303396	0.293398866	-1.200172035	2
381	BHD	0.413017868	0	0.646183419	4
382	CAN	0.412417164	0.257873136	-0.980478491	2
383	GOA	0.412363018	0	0.642062227	4
384	MME	0.412042443	0	0.640044743	4
385	TRD	0.411411461	0	0.636073761	4
386	MPL	0.410258782	0	0.62881957	4
387	OVD	0.409629862	0	0.624861568	4
388	GUM	0.409577055	0.278037037	-1.125250384	2
389	SSA	0.408612188	0.426987654	-2.068718426	1
390	VGO	0.407947013	0	0.614270837	4
391	HAN	0.407880056	0.297855372	-1.260653545	2
392	TRS	0.407599454	0	0.612083535	4
393	CCU	0.40374626	0.281024691	-1.18074782	2
394	HYD	0.403050939	0.260839506	-1.058091618	2
395	LCG	0.399505896	0	0.561148082	4
396	TOS	0.396632475	0	0.543064686	4
397	PEN	0.395324211	0.280969136	-1.233400951	2
398	BOO	0.394138644	0	0.527370176	4
399	SZX	0.373210484	0.197008601	-0.844178402	2
400	NKG	0.36372967	0.177492063	-0.781020253	2
401	SYX	0.361187138	0.199890261	-0.937980564	2
402	XMN	0.356439359	0.158442734	-0.707016839	2
403	TSN	0.354600016	0.168962351	-0.784795883	2
404	REC	0.349515888	0.349246914	-1.951382758	1
405	BNE	0.347291894	0.160208333	-0.775696383	2
406	MHD	0.346263668	0.20428125	-1.059532886	2
407	CTS	0.343855871	0.152777778	-0.750557459	2
408	FOR	0.341821651	0.33465374	-1.907965448	1
409	MFM	0.339709248	0.140268861	-0.697930764	2
410	PUS	0.33681054	0.144970414	-0.745761735	2
411	SHE	0.335151558	0.121321865	-0.607374072	2
412	CGK	0.332858089	0.121216049	-0.621141699	2
413	GOI	0.332434237	0.146708333	-0.784240571	2
414	KMQ	0.328101353	0.18	-1.021024393	2
415	URC	0.327274544	0.120025712	-0.648789616	2
416	DPS	0.311506246	0.109722222	-0.683181475	2
417	NAT	0.311477042	0.29722314	-1.863370957	1
418	CWB	0.302522944	0.269135734	-1.742958674	1
419	TNA	0.299811808	0.058769513	-0.436116111	2
420	CTU	0.299080699	0.04875	-0.377661087	2
421	DLC	0.299063307	0.04875	-0.377770543	2
422	PER	0.29013716	0.052930748	-0.460256595	2
423	KMG	0.281539496	0.001865702	-0.192995229	2
424	COK	0.278033828	0.004686981	-0.232812807	2
425	TAO	0.27771705	0	-0.205309669	2
426	HGH	0.277348942	0	-0.207626291	2
427	CKG	0.277118376	0	-0.209077322	2
428	TRV	0.276465346	0.005302469	-0.246557262	2
429	XIY	0.276438758	0	-0.213354385	2
430	WUH	0.276080816	0	-0.215607029	2
431	KWL	0.275623604	0	-0.218484418	2
432	HAK	0.275453391	0	-0.219555626	2
433	FOC	0.274909452	0	-0.222978816	2
434	CGO	0.274813405	0	-0.223583271	2
435	CSX	0.274673622	0	-0.224462972	2
436	FUK	0.274397759	0	-0.226199068	2
437	KWE	0.274190433	0	-0.227503842	2
438	TYN	0.273730836	0	-0.230396236	2
439	HRB	0.273718277	0	-0.230475275	2
440	WNZ	0.272839124	0	-0.236008077	2
441	LHW	0.272378391	0	-0.238907622	2

442	DUR	0.272214021	0.022125	-0.379182051	2
443	NGB	0.27212233	0	-0.240519101	2
444	CGQ	0.271720238	0	-0.243049601	2
445	SHA	0.271378811	0	-0.245198317	2
446	NNG	0.269944841	0	-0.254222763	2
447	OKA	0.268918822	0	-0.260679844	2
448	YNT	0.268271702	0	-0.264752388	2
449	HND	0.265534199	0	-0.281980401	2
450	KHH	0.264611624	0	-0.287786474	2
451	SDJ	0.262595336	0	-0.300475649	2
452	CJU	0.260387426	0	-0.314370756	2
453	ADL	0.26036022	0.001183432	-0.321989703	2
454	BKI	0.26023124	0	-0.315353686	2
455	CNS	0.26018389	0.001467456	-0.324886863	2
456	GAU	0.260033118	0.011944444	-0.391770901	2
457	PNQ	0.25911161	0.008493827	-0.375854372	2
458	RGN	0.257954748	0.01376	-0.41627667	2
459	CEB	0.257832313	0.007107438	-0.375180407	2
460	HIJ	0.257636372	0	-0.331684059	2
461	PNH	0.257461771	0	-0.332782876	2
462	ITM	0.256430149	0	-0.339275221	2
463	KOJ	0.251296939	0	-0.37158022	2
464	CNX	0.250035427	0	-0.379519335	2
465	DRW	0.250005095	0.009728395	-0.440934254	2
466	CHC	0.249438717	0.010246914	-0.447761865	2
467	KCH	0.245937096	0	-0.405311498	2
468	KMI	0.245847869	0	-0.405873028	2
469	NGS	0.245805876	0	-0.406137306	2
470	OOL	0.245761128	0.000277778	-0.408167068	2
471	SUB	0.245195239	0	-0.40998025	2
472	KMJ	0.244762453	0	-0.412703915	2
473	UKB	0.244662042	0	-0.413335835	2
474	MYJ	0.244644245	0	-0.413447838	2
475	WLG	0.243958954	0.000244898	-0.419301827	2
476	CBR	0.2371014	0.00048	-0.463938273	2
477	BEL	0.223963816	0.111122449	-1.242927221	1
478	MAO	0.221339668	0.099210938	-1.18447875	1
479	BSB	0.220491664	0.094905	-1.162716823	1
480	PLZ	0.21769923	0.009777778	-0.644556598	2
481	HBA	0.207213939	0.000375	-0.651369214	2
482	ISG	0.206673859	0	-0.652408113	2
483	HKD	0.205542433	0	-0.659528557	2
484	MES	0.203874453	0	-0.670025708	2
485	CGH	0.177394761	0	-0.836671222	1
486	POA	0.175706276	0	-0.847297425	1
487	CNF	0.172988394	0	-0.864401959	1
488	SLZ	0.166598674	0	-0.904614593	1
489	CPQ	0.166475257	0	-0.905391299	1
490	FLN	0.166394732	0	-0.905898071	1
491	VIX	0.163755796	0	-0.922505773	1
492	CGB	0.161801185	0	-0.93480679	1
493	GYN	0.158107259	0	-0.958053895	1
494	GMP	0.15246357	0	-0.993571507	2
495	UPG	0.151232581	0	-1.001318531	2
496	MZG	0.130842854	0	-1.129637872	2
497	TSA	0.1304326	0	-1.132219737	2
498	DMK	0.130257917	0	-1.133319072	2
499	SDU	0.001	0	-1.946782169	1
